# c-Abl Kinase Is Required for Satellite Cell Function Through Pax7 Regulation

**DOI:** 10.3389/fcell.2021.606403

**Published:** 2021-03-11

**Authors:** Fabián Montecino, Natalia González, Natasha Blanco, Manuel J. Ramírez, Adrián González-Martín, Alejandra R. Alvarez, Hugo Olguín

**Affiliations:** ^1^Laboratory of Tissue Repair and Adult Stem Cells, Department of Molecular and Cell Biology, Faculty of Biological Sciences, Pontificia Universidad Católica de Chile, Santiago, Chile; ^2^CARE-UC, Department of Molecular and Cell Biology, Faculty of Biological Sciences, Pontificia Universidad Católica de Chile, Santiago, Chile

**Keywords:** c-Abl, pax7, satellite cells, muscle stem cells, MRFs, skeletal muscle regeneration, muscle differentiation

## Abstract

Satellite cells (SCs) are tissue-specific stem cells responsible for adult skeletal muscle regeneration and maintenance. SCs function is critically dependent on two families of transcription factors: the paired box (Pax) involved in specification and maintenance and the Muscle Regulatory Factors (MRFs), which orchestrate myogenic commitment and differentiation. In turn, signaling events triggered by extrinsic and intrinsic stimuli control their function via post-translational modifications, including ubiquitination and phosphorylation. In this context, the Abelson non-receptor tyrosine kinase (c-Abl) mediates the activation of the p38 α/β MAPK pathway, promoting myogenesis. c-Abl also regulates the activity of the transcription factor MyoD during DNA-damage stress response, pausing differentiation. However, it is not clear if c-Abl modulates other key transcription factors controlling SC function. This work aims to determine the role of c-Abl in SCs myogenic capacity via loss of function approaches *in vitro* and *in vivo*. Here we show that c-Abl inhibition or deletion results in a down-regulation of Pax7 mRNA and protein levels, accompanied by decreased Pax7 transcriptional activity, without a significant effect on MRF expression. Additionally, we provide data indicating that Pax7 is directly phosphorylated by c-Abl. Finally, SC-specific c-Abl ablation impairs muscle regeneration upon acute injury. Our results indicate that c-Abl regulates myogenic progression in activated SCs by controlling Pax7 function and expression.

## Introduction

Skeletal muscle has a remarkable capacity to regenerate in response to injury or pathologic conditions (Olguín and Pisconti, 2012). This capability rests fundamentally in a group of tissue-specific stem cells called Satellite Cells (SCs). These cells are typically maintained in a quiescent state (G_0_) localized at the myofiber periphery, between the sarcolemma and the basal lamina (Muir et al., 1965; Hawke and Garry, 2001). In response to acute muscle damage, SCs are activated and re-enter the cell cycle. After a robust proliferation period, muscle progenitors can fuse to form a new myofiber or an injured muscle fiber to regenerate tissue function (Partridge, 2002).

Among other critical regulators, the transcription factor Pax7 controls the myogenic specification, and survival of SCs. In fact, in animal models where Pax7 is deleted, the number of SCs rapidly decay after birth, via apoptosis and early differentiation (Relaix et al., 2006; Buckingham and Relaix, 2007; von Maltzahn et al., 2013). On the other hand, Pax7 overexpression induces a quiescent-like state in muscle progenitors, linking its function to SC self-renewal (Olguin and Olwin, 2004). Moreover, high Pax7 levels inhibit MyoD function and myoblast differentiation (Olguin et al., 2007).

The Muscle Regulatory Factor (MRF) MyoD, is considered the master gene of myogenesis since its ectopic expression in non-myogenic cells results in the activation of the entire muscle differentiation program (Tapscott et al., 1988; Weintraub et al., 1989). Pax7 and MyoD co-expression are fundamental in the first hours after SC activation allowing lineage commitment and proliferation while avoiding precocious differentiation (Olguín and Pisconti, 2012). MyoD will eventually induce the expression of the MRF Myogenin, which directs myogenic progression to terminal differentiation (Hollenberg et al., 1993). Different mechanisms are involved in the post-translational regulation of Pax7 and the MRFs, including the ubiquitin-proteasome system, caspase-mediated proteolysis, and phosphorylation, among other mechanisms (Olguín, 2011; Bustos et al., 2015; Dick et al., 2015; González et al., 2016; de la Vega et al., 2020). In this context, the Abelson non-receptor tyrosine kinase 1 (c-Abl) regulates MyoD activity during DNA damage response (Simonatto et al., 2013). In that condition, c-Abl phosphorylates MyoD at the N-terminal domain (Tyr30), blocking MyoD-dependent gene expression, and pausing myogenesis until DNA is repaired (Puri et al., 2002; Innocenzi et al., 2011).

Early reports described different c-Abl expression levels in proliferative (high) and differentiated (low) muscle cells (Claycomb and Lanson, 1987). Recent work showed that c-Abl regulates differentiation in the muscle-derived cell line C2C12 by activating the p38 α/β MAPK pathway. c-Abl knock-down experiments in C2C12 cells resulted in decreased differentiation, reducing myoblasts fusion, and Myosin Heavy Chain (MyHC) expression (Bae et al., 2009). Besides, c-Abl exhibits differential subcellular localization in myoblasts (nuclear) and myotubes (cytoplasmic), suggesting that c-Abl could exert different functions during myogenesis, depending on differentiation status and its subcellular localization (Puri et al., 2002; di Bari et al., 2006).

Despite these observations, the role of c-Abl in the regulation of Pax7 and MRFs expression and function in adult muscle progenitors is unclear. Here we describe that c-Abl is required for myoblasts differentiation and proper muscle regeneration upon injury. We found that inhibition or deletion of c-Abl results in decreased Pax7 expression and transcriptional activity in activated SCs, which finally converges in poor differentiation and impaired muscle regeneration. Furthermore, we show that c-Abl interacts with and phosphorylates Pax7 protein. Together, these results suggest that c-Abl regulates SC function during muscle regeneration by controlling Pax7 expression and activity.

## Materials and Methods

### Cell Culture

Adult primary myoblasts and isolated myofibers were obtained as described (Cornelison et al., 2004) and maintained in proliferation medium, F-12C (Life technologies, United States) supplemented with 15% Horse Serum (HS) (Hyclone, United States) and 500 pM of FGF-2 at 37°C, 6% O_2_ and 5% CO_2_. When required, the cells were induced to differentiate by replacing the proliferation medium with differentiation medium, F-12C, supplemented with 15% HS. For *in vitro* recombination experiments, primary myoblasts were treated with 10 μM Tamoxifen T-5648 (Sigma-Aldrich, United States) dissolved in ethanol 100% once every 24 h for 2 days.

C2C12 myoblasts were maintained in proliferation medium, DMEM (Thermo Scientific, United States) supplemented with 10% Fetal Bovine Serum (FBS) (Biological Industries, United States) at 37°C and 5% CO_2_. For differentiation experiments, proliferation medium was replaced by differentiation medium, DMEM, supplemented with 5% HS. Cells with two or more nuclei were considered myotubes. For c-Abl inhibition assays, C2C12 cells were treated with 10 μM Imatinib Mesylate (Sigma-Aldrich, United States) for the indicated time in every experiment. For c-Abl activation, cells were treated with DPH (Sigma-Aldrich, United States) for the indicated time and concentrations. DMSO (Sigma-Aldrich) was used as a vehicle.

### Immunofluorescence Staining

Initially, C2C12 cells were plated and maintained as described above. Primary myoblasts were seeded onto 0.66% gelatin-coated glass-slides and maintained as described previously. When specified, cells were fixed with 4% paraformaldehyde (PFA) for 10 min, then permeabilized with PBS 0.5% Triton X-100 (Sigma-Aldrich, United States) for 5 min and blocked with PBS 3% BSA (Sigma-Aldrich, United States) for 60 min and subjected to standard immunofluorescence (IF) staining (Bustos et al., 2015). The following primary antibodies and dilutions were used: mouse monoclonal anti-Pax7 1:1, anti-Myogenin (F5D) 1:2, and anti-MyHC (MF20) 1:1 (Hybridoma conditioned medium, Developmental Studies Hybridoma Bank, United States); rabbit polyclonal anti-c-Abl (K-12), 1:200 (Santa Cruz Biotechnology, United States); rabbit polyclonal anti-c-Abl, 1:250 (Cell Signaling, United States); mouse monoclonal anti-c-Abl (ABL-148), 1:250 (Sigma-Aldrich, United States); rabbit polyclonal anti-phospho-c-Abl (Tyr412), 1:250 (Sigma-Aldrich, United States); rabbit polyclonal anti-phospho-c-Abl (Tyr412), 1:100 (Millipore, United States); rat monoclonal anti-MyoD (5F11), 1:100 (Merck, United States); goat polyclonal anti-Myogenin (N-20), 1:500 (Santa Cruz Biotechnology, United States); and chicken anti-Syndecan-4, 1:500 (Cornelison et al., 2004). Secondary antibodies and dilutions were: donkey anti-mouse IgG Alexa 555; donkey anti-rabbit IgG Alexa 555; donkey anti-rabbit IgG Alexa 488; donkey anti-mouse IgG Alexa 488; donkey anti-rat IgG Alexa 555; donkey anti-goat IgG Alexa 555; donkey anti-rat IgG Alexa 488; and goat anti-chicken IgY Alexa 555, 1:500 (Life technologies, United States); 1 μg/mL of Hoechst 33342 (Sigma-Aldrich, United States) was added to nuclei counterstaining. Fluoromount (Sigma-Aldrich, United States) was used for mounting. Fluorescence was evaluated using a Motic BA410 Elite Trinocular microscope equipped with a refrigerated Moti-cam Pro 252B camera. Images were acquired using the MOTIC IMAGES PLUS 3.0 software. Image analysis were performed using ImageJ software (Schneider et al., 2012).

### Mice Strains and *in vivo* Tamoxifen-Induced Recombination and Muscle Injury

For conditional c-Abl deletion in SCs, we generated C57BL/6J *Abl1*^flox/flox^-Pax7^creERT2^ transgenic mouse line (from JAX stock #013224 and #017763, Jackson Laboratories) (Murphy et al., 2011). To induce recombination, mice were injected via intraperitoneal with 1 mg of Tamoxifen T-5648 (Sigma-Aldrich, United States) dissolved in sesame oil with 5% Ethanol (Sigma-Aldrich, United States) per day, for 5 consecutive days. Seventy two hours after the last tamoxifen dose, muscle injury was performed as described previously (Cornelison et al., 2004). Briefly, *Tibialis anterior* (TA) muscles from 12 weeks old male mice were injected with 50 μL of 1.2% of barium chloride (BaCl_2_) diluted in saline (0.9% NaCl); contralateral TAs were injected with 50 μL of saline as control. TA muscles were extracted at 7-, 15-, and 30-days post-injury (dpi) and processed for histological analyses, IF, or lysis. For *in vitro* SCs tracing *Abl1*^flox/flox^-Pax7^creERT2^ were bred with B6.129(Cg)*-Gt(ROSA)26Sor^TM 4(ACTB–tdTomato,–EGFP)Luo/J^* (ROSA^mT/mG^) reporter mice, obtained from Jackson Laboratories (JAX stock #007576). This model allows us to identify recombinant cell populations by the expression of cell membrane-localized EGFP (mGFP), in contrast with the expression of membrane-tdTomato (mTomato) in all non-recombinant cells. Only Pax7 expressing cells going under recombination.

All animal procedures were performed according to National Commission for Science and Technology (CONICYT) guidelines and approved by the School of Biological Sciences and the Pontificia Universidad Católica de Chile Bioethics and Biosecurity Committee (protocol ID 160929002).

### Muscle Tissue Staining

*Tibialis anterior* (TA) muscles from 8–12 weeks old male C57BL/6J mice were dissected, and snap-frozen on liquid nitrogen chilled isopentane (Sigma-Aldrich, United States), transverse cryosectioned (7 μm) and subjected to IF. Antigen retrieval was performed before Pax7 IF as described previously (Hussaini et al., 2013). Following primary antibodies and dilutions were used: chicken anti-laminin, 1:2000 (Sigma-Aldrich, United States), rat monoclonal anti-MyoD (5F11), 1:500 (Merck, United States); rabbit polyclonal anti-phospho-Abl (Tyr412), 1:100 (Millipore, United States); mouse monoclonal anti-Pax7, 1:1 (Hybridoma conditioned medium, Developmental Studies Hybridoma Bank, United States); rabbit monoclonal anti-ki67 (SP6), 1:100 (Abcam, United Kingdom); and goat polyclonal anti-Myogenin (N-20), 1:250 (Santa Cruz Biotechnology, United States). Secondary antibodies and dilutions were donkey anti-rabbit IgG Alexa 488; donkey anti-mouse IgG Alexa 647; goat anti-chicken IgY Alexa 555; donkey anti-rat IgG Alexa 647; donkey anti-goat IgG Alexa 647; and donkey anti-rat IgG Alexa 555 at 1:500 (Thermo Scientific, United States); 1 μg/mL of Hoechst 33342 was added to nuclei counterstaining. Fluoromount (Sigma-Aldrich, United States) was used for mounting. For Hematoxylin/Eosin (H&E) staining, sections were fixed with 4% PFA, rinsed with distilled water, and stained with hematoxylin for 5 min. Then, sections were washed with tap water and stained with eosin for 1 min. Sections were dehydrated on an ascending ethanol concentration battery and mounted with Entellan (Sigma-Aldrich, United States).

### Western Blotting and Co-immunoprecipitation

Cells and tissue were lysed in modified RIPA buffer (50 mM Tris-HCl pH 7.4, 150 mM NaCl, 1% IGEPAL), supplemented with Protease Inhibitor Cocktail Set III (Merck, United States) and phosphatase inhibitors (1 mM sodium orthovanadate, 5 mM sodium fluoride, 1 mM β-glycerophosphate, and 1 mM pyrophosphate). 10–30 μg of total protein was loaded into 10% SDS-Polyacrylamide gel electrophoresis (PAGE) gels and transferred to polyvinylidene fluoride (PVDF) membranes. Western blotting (WB) was performed with the following primary antibodies and dilutions: mouse monoclonal anti-Pax7, anti-MyHC (MF20) and anti-Myogenin (F5D), 1:5 (Hybridoma conditioned medium, Developmental Studies Hybridoma Bank, United States); mouse monoclonal anti-c-Abl (24-11), 1:1000 (Santa Cruz Biotechnology, United States); rabbit polyclonal anti-c-Abl, 1:2000 (Cell Signaling, United States); mouse monoclonal anti-c-Abl (ABL-148), 1:2000 (Sigma-Aldrich, United States); rabbit polyclonal anti-phospho-Abl (Tyr412), 1:1000 (Millipore, United States); rat monoclonal anti-MyoD (5F11), 1:1000 (Merck, United States); mouse monoclonal anti-myc-tag (9B11), 1:5000 (Cell Signaling, United States); mouse monoclonal anti-α-Tubulin (B-5-1-2), 1:20000 (Sigma-Aldrich, United States); mouse monoclonal anti-Gapdh (6C5), 1:10000 (Millipore, United States); and mouse monoclonal anti-phosphotyrosine (4G10), 1:2500 (Millipore, United States). Secondary antibodies used were HRP conjugated: anti-mouse IgG (Jackson ImmunoResearch, United States), anti-rabbit IgG and anti-rat IgG (Thermo Scientific, United States), at 1:5000. HRP activity was detected using the Westar ECL Substrates (Cyanagen, Italy).

For immunoprecipitation (IP), Dynabeads Protein G (Thermo Scientific, United States) was used according to the manufacturer’s instructions. Briefly, 30 μL of Dynabeads Protein G was incubated with 200 μL of mouse monoclonal anti-Pax7 (Hybridoma conditioned medium, Developmental Studies Hybridoma Bank, United States) or normal mouse IgG (Santa Cruz Biotechnology, United States) at 1:200; for 1 h 30 min at room temperature. Then, total protein was equalized (∼500 μL at 1 mg/mL, 10% was loaded as input) and then incubated with Dynabeads-antibody complexes for 2 h at room temperature. Proteins were eluted by resuspending Dynabeads in 30 μL 2X SDS-PAGE loading buffer and boiled for 5 min.

### *In vitro* Phosphorylation Assay

Purified GST-Pax7 Full length (5 μg) and GST-MyoD (1 μg) were incubated with or without 10 ng of recombinant c-Abl His-Tag (ENZO life sciences, United States) in kinase buffer (MOPS 25 mM pH 7.2, 12.5 mM β-glycerophosphate, 25 mM MgCl_2_, 325 μM EGTA, 2 mM EDTA, 250 μM DTT) supplemented with 100 μM ATP (New England Biolabs, United States) for 60 min at 30°C. Two hundred and fifty μM of Imatinib Mesylate (Sigma-Aldrich, United States) or Lambda Protein Phosphatase (New England Biolabs, United States) were added as indicated. Reactions were loaded into SDS-PAGE gel and analyzed by WB.

### Pax7 Phosphorylation in C2C12 Cells Experiment

C2C12 cells were treated with vehicle, 10 μM Imatinib or DPH, for 24 h and 10 μM pervanadate was added to the medium 1 h prior to lysis, except in non-treated cells (N/T). Pervanadate acts as an inhibitor of protein tyrosine phosphatases (PTPs), allowing the accumulation of phospho-tyrosine proteins (Huyer et al., 1997). Pervanadate was prepared using sodium orthovanadate and hydrogen peroxide as previously described (Gray et al., 2014). Pax7 was immunoprecipitated under denaturing conditions in order to avoid pull-down of its interacting proteins. For this, denaturing lysis of C2C12 cells was performed using modified RIPA buffer supplemented with 1% sodium deoxycholate and 0.1% sodium dodecyl sulfate (SDS), plus protease and phosphatase inhibitors mentioned before. IP was performed as previously described. In order to achieve greater separation of immunoprecipitated Pax7 proteins, a 8% SDS-Polyacrylamide gel was used in electrophoresis. WB against phospho-tyrosine was executed in order to detect phospho-tyrosine Pax7 that were immunoprecipitated.

### Real-Time PCR

C2C12 cells treated with vehicle or Imatinib 10 μM for 6, 18, and 24 h, were lysed in TRIzol reagent according to the manufacturer’s instructions (Invitrogen, United States) and total RNA was quantified using NanoQuant plate of TECAN Infinite M200 PRO microplate reader; 500 ng of total RNA was retro-transcribed with M-MLV-RT enzyme (Promega, United States) and Random Hexamer Primer (Thermo Scientific, United States), according to the manufacturer’s instructions. qPCR reactions were performed with SYBR Green PCR master mix (Thermo Scientific, United States), following the manufacturer’s instructions on QuantStudio 3 Real-Time PCR System (Applied Biosystems, United States) with the following oligonucleotides: Pax7 Fwd: 5′-CACCCCTTTCAAAGACCAAA-3′, Pax7 Rev: 5′-TGCTTGAAGTTCCTGCTCCT-3′; and Gapdh as housekeeping gene, Gapdh Fwd: 5′-AGGTCGGTGTGAACGGATTTG-3′; Gapdh Rev: 5′-TGTAGACCATGTAGTTGAGGTCA-3′.

### Reporter Gene Assay

To evaluate Pax7 transcriptional activity when c-Abl is inhibited, C2C12 cells were transfected with the Pax3/7 specific reporter gene 6xPRS9-Luc (Olguin et al., 2007) and pcDNA3-myc-Pax7WT. CMV-LacZ vector for constitutive β-galactosidase expression was co-transfected in order to normalize luciferase activity. Pax7-FKHR was used as positive control for reporter activation, as described previously (Bennicelli et al., 1999; Olguin et al., 2007) and CMV empty vector as a negative control. Twenty four hours post-transfection, cells were treated with DMSO as vehicle or Imatinib 10 μM for 18 h. Finally, whole-cell lysates were collected, and luciferase and β-galactosidase activities were determined using the Dual-Light System (Applied Biosystems, United States) following the manufacturer’s instructions. Luminescence was measured in TECAN Infinite M200 PRO microplate reader.

### Statistics

All quantitative data are presented as mean ± SEM unless indicated. The number of independent experiments is specified in figure legends. Statistical analysis was performed with GraphPad Prism software, using the corresponding statistical test to determine significance. A *P*-value < 0.05 was considered significant.

## Results

### c-Abl Expression and Activation in Muscle Progenitors

**FIGURE 1:**
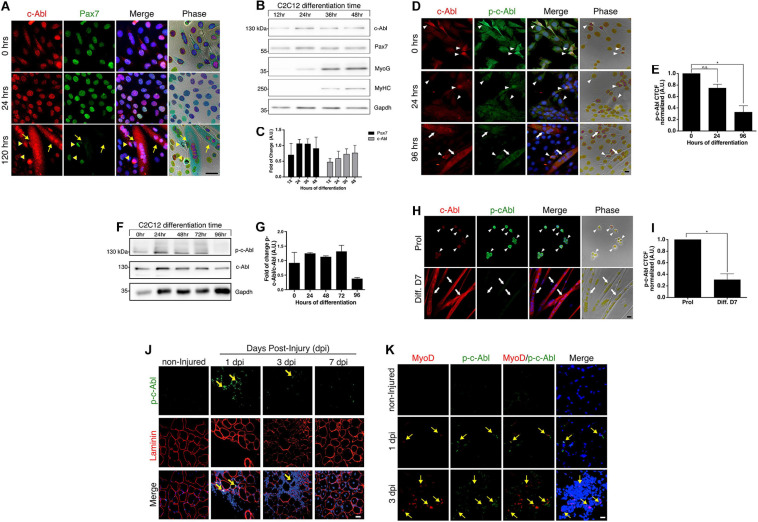
c-Abl is expressed in myoblasts and activated during muscle regeneration. **(A)** Pax7 and c-Abl nuclear expression are correlated. C2C12 cells were differentiated the indicated time and then fixed. IF was performed against c-Abl (red), Pax7 (green), and nuclei were stained with Hoechst 33342 (blue). Arrows show myotubes without Pax7 expression, while arrowheads show mononucleated cells expressing Pax7 and c-Abl. Scale bar: 10 μm. **(B)** Expression of Pax7 and c-Abl in early myoblast differentiation. C2C12 cells were maintained in differentiation conditions for the indicated times and lysed. WB against Pax7, c-Abl, Myogenin (MyoG), MyHC and Gapdh was performed. **(C)** Graph shows quantification of Pax7 and c-Abl protein levels from B (*n* = 4). A.U., arbitrary units. **(D)** Expression and phosphorylation of c-Abl in C2C12. Cells were induced to differentiate, and IF for c-Abl (red) and phospho-tyr412 c-Abl (p-c-Abl, green) was performed at the indicated times. Nuclei were stained with Hoechst 33342 (blue). Arrows show myotubes and arrowheads myoblasts. Scale bar: 10 μm. **(E)** Quantification of **(D)**. Graph shows quantification of Corrected Total Cell Fluorescence (CTCF) for p-c-Abl normalized to 0 h (*n* = 3, *P-value < 0.05, Kruskal–Wallis test, ns, not significant) A.U., arbitrary units. **(F)** Expression of p-c-Abl and c-Abl in myoblast differentiation. C2C12 cells were maintained in differentiation conditions for the indicated times and lysed. WB against p-c-Abl, c-Abl, and Gapdh was performed. **(G)** Graph shows quantification of Pax7 and c-Abl protein levels from F (*n* = 2). A.U., arbitrary units. **(H)** Expression and phosphorylation of c-Abl in primary myoblasts. Cells were maintained in proliferation conditions (Prol) or 7 days in differentiation conditions (Diff. D7), followed by IF for c-Abl (red) and p-c-Abl (green). Nuclei were stained with Hoechst 33342 (blue). Arrows show myotubes and arrowheads myoblasts. Scale bar: 10 μm. **(I)** Quantification of **(H)**. Graph shows quantification of CTCF for p-c-Abl normalized to proliferation (*n* = 3, *P-value < 0.05, Wilcoxon signed-rank test). A.U., arbitrary units. **(J)** c-Abl is transiently phosphorylated in early muscle regeneration. Sections of muscles were obtained at 0 (non-injured), 1, 3, and 7 dpi, fixed and IF for p-c-Abl (green) and laminin (red) was performed. Nuclei were stained with Hoechst 33342 (blue). Yellow arrows show p-c-Abl in cells associated with laminin. Scale bar: 50 μm. **(K)** Myogenic cells are positive for p-c-Abl. Muscle cryosections were obtained at 0 (non-injured), 1, and 3 dpi, fixed and IF for p-c-Abl (green), and MyoD (red) was performed. Nuclei were stained with Hoechst 33342 (blue). Yellow arrows show MyoD positive cells with expression of p-c-Abl. Scale bar: 50 μm.

It has been described that c-Abl shuttles between the nucleus and cytoplasm in myoblasts and myotubes (Puri et al., 2002). We corroborated these findings, and additionally, we observed a correlation between c-Abl localization and Pax7 levels in C2C12 cells. High Pax7 expression was accompanied by nuclear c-Abl localization (Figure 1A, 0 and 24 h). As expected, Pax7 expression was not detected in myotubes, where c-Abl was also observed in a cytoplasmic localization (Figure 1A, 120 h and Supplementary Figure 1A). This result suggests a correlation between Pax7 expression and c-Abl localization during the differentiation of C2C12 cells. Western blot analyses showed that c-Abl expression remains unchanged after 48 h in differentiating culture conditions (Figures 1B,C). Expression of the differentiation markers Myogenin and Myosin heavy chain (MyHC), can be detected from 24 and 36 h, respectively (Figure 1B). As described previously (Bustos et al., 2015), Pax7 protein remains detectable during this period (Figures 1B,C), likely to non-differentiating cells.

We evaluated c-Abl expression and activation status in C2C12 cells maintained in proliferation (Figure 1D, 0 h) and differentiation culture conditions (Figure 1D, 24 and 96 h). As previously described (Brasher and Van Etten, 2000), c-Abl activation was determined by detecting phosphorylation at tyrosine 412 (phospho-Tyr412). Active c-Abl was detected mostly in myoblasts, while the level of phosphorylated c-Abl was significantly lower in differentiated, multinucleated cells (Figures 1D,E and Supplementary Figure 1B). Western blot analyses of whole-cell extracts obtained from C2C12 cells, also indicate a reduction in phosphorylated c-Abl levels after 96 hrs in differentiation conditions (Figures 1F,G).

c-Abl expression and activation status were also analyzed by IF in primary myoblasts, isolated from adult mice and maintained in proliferation and differentiation culture conditions for 7 days. Total c-Abl and phospho-c-Abl were detected in both conditions (Figure 1H), although phospho-c-Abl signal in multinucleated cells was significantly lower in comparison with proliferating cells (Figure 1I), similar to the distribution observed in C2C12 cells. Together, these results indicate that c-Abl kinase activity is differentially regulated in proliferating and differentiating muscle progenitors.

### c-Abl Is Activated During Early Muscle Regeneration

To determine c-Abl expression and activation dynamics during muscle regeneration, acute injury was performed, by barium chloride (BaCl_2_) intramuscular injection, in the *Tibialis Anterior* (TA) muscle from adult mice. Muscles were isolated at 1-, 3-, and 7-days post-injury (dpi) in order to analyze different stages of early regeneration by IF. The levels of c-Abl phosphorylation were determined as described before. No signal for phospho-Tyr412 was detected in uninjured muscles, neither in cells nor fibers (Figure 1J, non-injured panel). Phospho-c-Abl signal was detected in myogenic cells early after injury (Figure 1J, 1 and 3 dpi panels), defined by co-expression of MyoD (Figure 1K, 1 and 3 dpi panels). Phospho-c-Abl was also detected in non-myogenic cells, likely corresponding to infiltrating inflammatory cells (Figure 1K, 1 and 3 dpi panels). Interestingly, c-Abl activation appears to be transient, since phospho-c-Abl was not detected at 7 dpi (Figure 1J and Supplementary Figure 2A). These results indicate that c-Abl Tyr412 phosphorylation is also differentially regulated in myogenic progenitors *in vivo* and may play a role in muscle regeneration.

### c-Abl Inhibition Prevents Differentiation in C2C12 Cells

**FIGURE 2:**
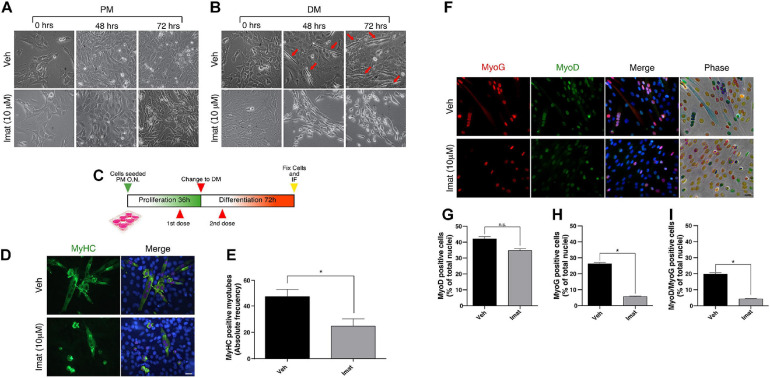
c-Abl inhibition impairs C2C12 cells differentiation. **(A,B)** c-Abl inhibition affects C2C12 cells with “myotube like” phenotype. C2C12 cells were maintained in proliferation medium (PM) or differentiation medium (DM) for 72 h in the presence of 10 μM of Imatinib (Imat) or vehicle (Veh) (added fresh every 24 h). Micrographs were obtained every 24 h to evaluate phenotypic changes in cells. Red arrows indicate C2C12 cells with “myotube like” phenotype. **(C)** Experimental strategy to evaluate C2C12 cells differentiation. Cells were seeded in PM overnight (O.N.) and then treated with vehicle or Imatinib for 24 h (1st dose). Next, PM was replaced by DM, and cells were treated again (2nd dose). After 72 h, cells were fixed, and IF for MyoD, Myogenin (MyoG) and MyHC was performed. Nuclei were stained with Hoechst 33342 (blue). **(D)** Long c-Abl inhibition impairs C2C12 cell differentiation. Cells treated twice with Imatinib show a decrease in the number of differentiated myotubes (cells with two or more myonuclei), in comparison with the vehicle. Scale bar: 10 μm. **(E)** Quantification of the number of MyHC positive myotubes from **(D)**. Veh mean: 47.0 ± 5.5 cells; Imat mean: 24.66 ± 5.6 (*n* = 3, *P-value < 0.05, unpaired *t*-student test. **(F)** c-Abl inhibition does not change MyoD but Myogenin expression in cells. Plots **(G,H)** show the percentage of MyoD and Myogenin positive cells treated as in **(F)**, respectively. (*n* = 3, *P-value < 0.05, Nested *t*-test, ns, not significant). Plot **(I)** shows the mean of MyoD/Myogenin double positive nuclei per myotube (*n* = 3, *P-value < 0.05, Nested *t*-test).

c-Abl expression, localization, and activation patterns suggest distinct roles in muscle progenitors during their myogenic progression. Therefore, we tested the effects of c-Abl inhibition in C2C12 myoblasts. For this, we used Imatinib Mesylate which acts as a competitive inhibitor for c-Abl kinase activity (Schindler, 2000). Cells were maintained in proliferation (PM) or differentiation (DM) culture conditions for 72 h; in the presence of Imatinib or vehicle. Phase-contrast microscopy revealed no significant changes in cell morphology between treated and untreated cells maintained in PM (Figure 2A). However, Imatinib treatment resulted in a significant reduction in cell fusion and myotube formation both at 48 and 72 h in DM, compared to vehicle (Figure 2B). These observations agree with previous reports, which showed that c-Abl loss of function impairs myoblasts differentiation (Bae et al., 2009).

Additionally, we expose the cells to vehicle or Imatinib using a two-dose regimen treatment: first during the proliferation phase, followed by a second dose during the differentiation phase (Figure 2C). Cells were then fixed, and MyHC expression was analyzed by IF (Figure 2D). We observed a decrease in the number of MyHC-positive myotubes in cells treated twice with Imatinib (∼50%) compared to the vehicle (Figure 2E, *P*-value < 0.05). We then studied the effect of c-Abl inhibition on the expression of early differentiation markers such as MyoD and Myogenin (Figure 2F). We detected no significant differences on MyoD expression between vehicle and Imatinib treated cells (Figure 2G). Interestingly, the percentage of Myogenin expressing cells decreased by ∼5 fold upon Imatinib treatment (Figure 2H; *P*-value > 0.05). The percentage of MyoD/Myogenin expressing cells was similarly affected by Imatinib treatment (Figure 2I; *P*-value > 0.05).

Taken together these results indicate that c-Abl kinase activity is required for C2C12 cells differentiation. Since c-Abl inhibition did not affect MyoD expression, we hypothesized that c-Abl activity could regulate myogenesis up-stream of Myogenin induction in muscle progenitors.

### c-Abl Inhibition Results in Decreased Pax7 Levels

**FIGURE 3:**
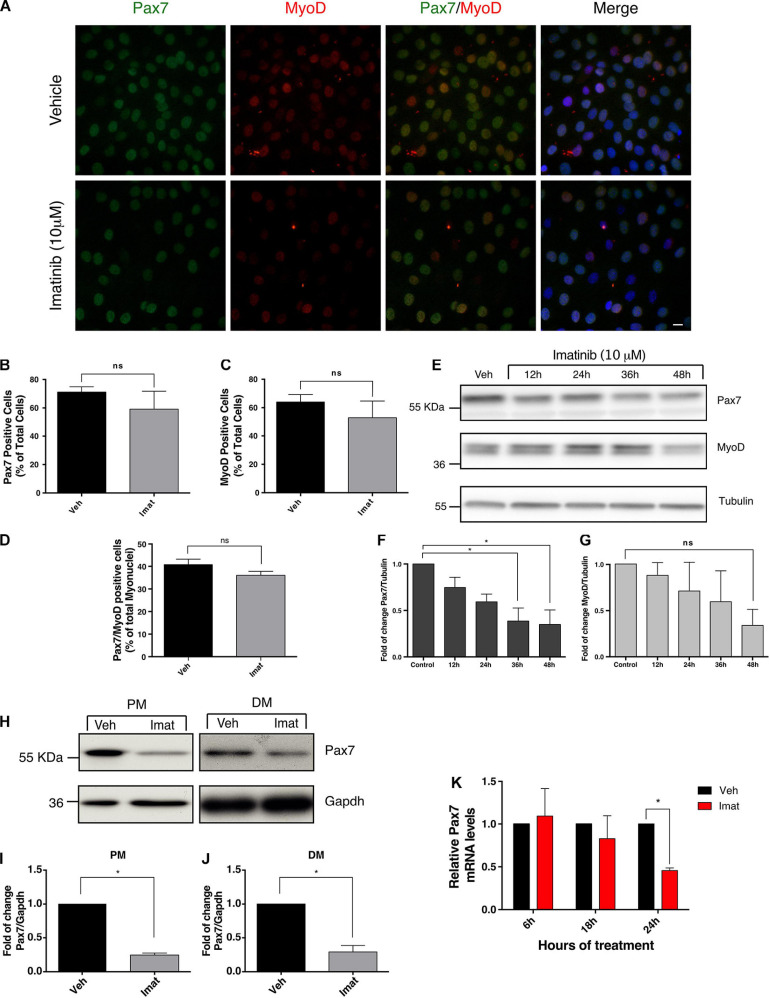
c-Abl inhibition decreases Pax7 protein and mRNA levels. **(A)** Imatinib treatment does not decrease the number of Pax7 or MyoD positive cells. C2C12 cells were maintained in PM and treated with vehicle (Veh) or 10 μM Imatinib (Imat) for 48 h. Then cells were fixed, and IF for Pax7 (green) and MyoD (red) was performed. Nuclei were stained with Hoechst 33342 (blue). Scale bar: 10 μm. **(B)** Quantification of Pax7 positive cells from **(A)**. Veh mean: 71.11 ± 3.77%; Imat mean: 59.16 ± 12.62%. **(C)** Quantification of MyoD positive cells from **(A)**. Veh mean: 64.05 ± 5.16; Imat mean: 52.97 ± 11.70%. **(D)** Quantification of Pax7/MyoD double positive cells from **(A)**. Veh mean: 40.79 ± 2.38%; Imat mean: 36.06 ± 1.75% (*n* = 4, *P*-value > 0.05, Mann–Whitney test, ns, not significant). **(E)** Imatinib induces a time-dependent drop of Pax7 protein. C2C12 cells were maintained in PM as specified and treated with 10 μM Imatinib every 24 h. Cells were lysed, and WB was performed to determine levels of Pax7 and MyoD. Tubulin was used to normalize protein levels. Plots **(F,G)** show Pax7 and MyoD fold of change from **(E)** respectively (*n* = 3, *P-value < 0.05, ANOVA test, ns: not significant). **(H)** c-Abl inhibition downregulates Pax7 protein levels in C2C12 cells. Cells were maintained in PM or DM for 48 h in the presence of vehicle or Imatinib (10 μM). Then cells were lysed, and WB against Pax7 was performed. Gapdh was used to normalize protein levels. Plots **(I,J)** show a significant decrease in Pax7 protein levels in cells treated with Imatinib versus vehicle (*n* = 4, *P-value < 0.05, Wilcoxon signed-rank test). **(K)** Pax7 mRNA levels decrease upon c-Abl inhibition. C2C12 cells in PM were treated with vehicle or Imatinib (10 μM) as indicated. A significant drop in Pax7 mRNA levels is observed at 24 h in Imatinib treated cells. Veh mean: 1.0 ± 0.0; Imat mean: 0.457 ± 0.03 (*n* = 3, *P-value < 0.05, multiple *t*-test).

To better understand the molecular basis of the differentiation impairment upon c-Abl inhibition, we evaluated the expression of Pax7 and MyoD proteins, which may reflect changes in the differentiation potential of myogenic progenitors. We performed IF to determine the percentage of cells expressing Pax7 and MyoD upon c-Abl inhibition (Figure 3A). C2C12 cells maintained in proliferation conditions were treated with vehicle or Imatinib for 48 h. We observed a small, but consistent, change in the percentage of cells expressing Pax7 after the treatment with Imatinib (59.16 ± 12.62%) in comparison with the vehicle (71.11 ± 3.77%), which did not reach statistical significance (Figure 3B). A similar trend was observed for MyoD expressing cells, with an average of 64.05 ± 5.16% and 52.97 ± 11.70% for vehicle and Imatinib, respectively (Figure 3C). The percentage of double positive cells for Pax7 and MyoD remain unchanged upon Imatinib treatment (Figure 3D). Additionally, Imatinib treatment in myofiber associated SCs for 48 h, did not alter the number of MyoD positive cells (Supplementary Figure 3). Although the percentage of Pax7, MyoD, or Pax7/MyoD expressing cells did not change significantly, we detected a consistent decrease in fluorescent signal upon Imatinib treatment. Therefore, Pax7 and MyoD protein levels were evaluated by Western blot (Figure 3E). We observed a significant time-depend decay of Pax7 protein levels but not in MyoD, reaching a larger decrease at 48 h of treatment (Figures 3F,G). Moreover, Imatinib treatment induced a significant Pax7 decrease (∼4-fold reduction compared to vehicle) in both proliferating and differentiating conditions (Figures 3H–J). Accordingly, treatment of C2C12 cells with the c-Abl activator DPH (Yang et al., 2011), resulted in a robust increase in Pax7 but not in MyoD or Myogenin protein levels (Supplementary Figure 4). These results strongly suggest that Pax7 expression is regulated by c-Abl activity.

To determine whether Pax7 down-regulation induced by Imatinib is due to transcriptional changes, we performed quantitative Real-Time Polymerase Chain Reaction (qRT-PCR). C2C12 cells were treated with vehicle or Imatinib for 6, 18, or 24 h, followed by lysis and total RNA isolation. As shown in Figure 3K, no significant changes in Pax7 mRNA levels were detected at 6 or 18 h of Imatinib treatment compared to vehicle. However, a significant decrease was observed at 24 h. This Pax7 transcript drop precedes the decrease in the protein levels analyzed above, which is noted from 36 h after Imatinib treatment (Figure 3E). These results indicate that c-Abl kinase activity regulates Pax7 expression, affecting the outcome of muscle differentiation.

### c-Abl Interacts With Pax7 in Myogenic Cells

**FIGURE 4:**
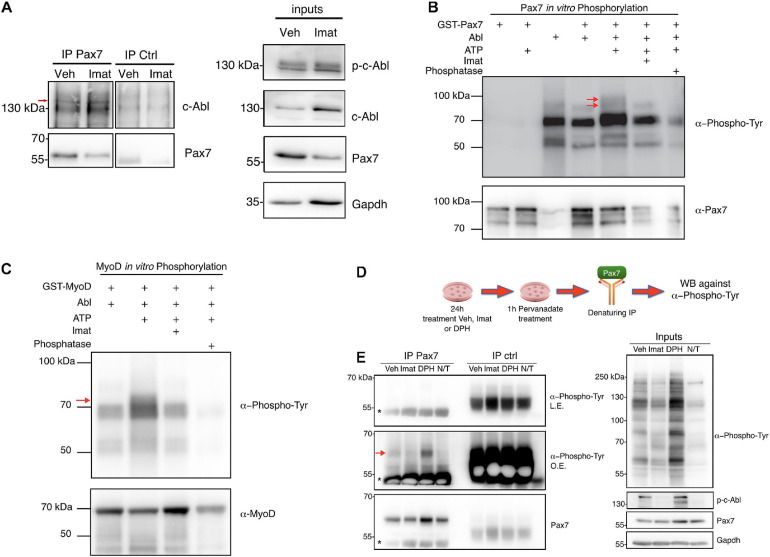
c-Abl interacts and phosphorylates Pax7. **(A)** c-Abl co-immunoprecipitates with Pax7 in C2C12 cells. Cells maintained in PM were treated with vehicle (Veh) or 10 μM Imatinib (Imat) for 24 h, followed by lysis and immunoprecipitation (IP) with anti-normal mouse IgG (IP ctrl) or anti-Pax7 (IP Pax7). c-Abl and Pax7 were detected by Western blot (Left panels). Red arrow shows the expected c-abl relative migration. Right panels show inputs for p-c-Abl, c-Abl, Pax7, and Gapdh (*n* = 3). **(B)** Pax7 is phosphorylated by c-Abl. Recombinant c-Abl His-Tag and GST-Pax7 were incubated in kinase buffer as indicated, followed by WB for Phospho-Tyr and Pax7. Red arrows show Pax7 phosphorylated in tyrosine residues (*n* = 3). As positive control, GST-MyoD was incubated with c-Abl, as is shown in **(C)**. Red arrow shows MyoD phosphorylated in tyrosine residues (*n* = 3). **(D,E)** Pax7 is phosphorylated by c-Abl in C2C12 cells. The figure shows the following protocol to determine Pax7 phosphorylation in C2C12. Left panels show Pax7 IP followed by WB against Phospho-Tyr and Pax7 (L.E., less exposed membrane; O.E., overexposed membrane). Red arrow shows Pax7 phosphorylated in tyrosine residues and asterisk indicates IgG. Right panels show inputs for Phospho-Tyr, p-c-Abl, Pax7, and Gapdh (*n* = 3).

Considering (i) the effects of c-Abl inhibition on Pax7 expression, and (ii) c-Abl nuclear localization in Pax7-expressing cells, we hypothesized that c-Abl and Pax7 physically interact in muscle progenitors. With this in mind, we performed co-immunoprecipitation (co-IP) assays on C2C12 cells (see section “Materials and Methods”). First, we tested c-Abl and Pax7 interaction in C2C12 cells treated with vehicle or Imatinib for 24 h, followed by Pax7 IP and c-Abl detection by Western blot. Under these conditions, there was an enrichment in c-Abl co-IP, compared to control IP with a non-related antibody (Figure 4A, left panel). This enrichment was detected both in cells treated with vehicle or Imatinib, despite the decrease in Pax7 levels, suggesting that c-Abl could interact with Pax7 independently of its activation status.

Considering the kinase nature of c-Abl, we performed *in vitro* phosphorylation assays as described previously (González et al., 2016), using recombinant c-Abl His-Tag and GST-Pax7, followed by Western blot to detect phosphorylated tyrosine residues (see section “Materials and Methods”). We observed two distinct bands, just under 100 kDa, coinciding with GST-Pax7 molecular weight (Figure 4B, red arrows). Importantly, these bands were only detected when GST-Pax7, c-Abl, and ATP were present in the reaction. Moreover, these phospho-proteins were not detected when Imatinib was added or when either ATP or c-Abl were omitted from the reaction mix (Figure 4B). Phosphatase treatment prior to Western blot had the same effect, further supporting the phosphoprotein nature of the bands identified above. Since MyoD has been described as a c-Abl substrate (Puri et al., 2002; Innocenzi et al., 2011), we used GST-MyoD as a positive control for the phosphorylation assay (Figure 4C). GST-only was used as an additional negative control, and no phospho-bands were observed nearby Pax7 molecular weight (Supplementary Figure 5).

To determine whether c-Abl phosphorylates Pax7 in a cellular context, we performed a denaturing IP of Pax7 in C2C12 cells treated with vehicle, Imatinib or DPH for 24 h. Western blot was performed to analyze the presence of phosphorylated tyrosine residues in Pax7. Given the rapid turnover of tyrosine phosphorylation in the cell environment, we inhibited PTPs using pervanadate 1 h prior to lysis (Figure 4D). Pervanadate treatment allows accumulation of phospho-tyrosine proteins compared to non-treated cells (Figure 4E, right panel, Veh versus N/T). Under these conditions, a phospho-tyrosine signal was detected in Pax7 IP from vehicle-treated extracts, which notably increased upon c-Abl activation (DPH treatment). Almost no signal was observed in Imatinib-treated extracts, or when pervanadate treatment was omitted (Figure 4E, left panel, red arrow).

Together, these results indicate that Pax7 and c-Abl physically interact in muscle progenitors and suggest that Pax7 is a c-Abl kinase substrate in myogenic cells.

### c-Abl Inhibition Impairs Pax7 Transcriptional Activity

**FIGURE 5:**
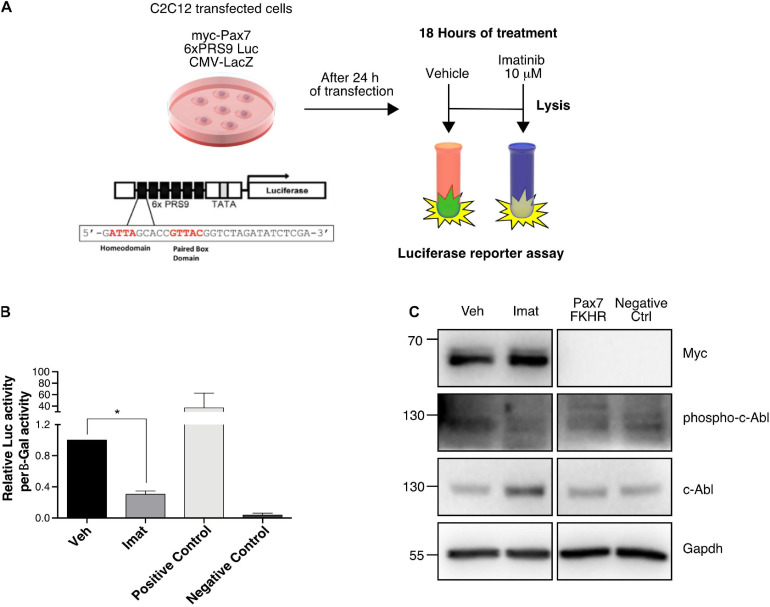
c-Abl regulates Pax7 transcriptional activity. **(A)** Protocol for Reporter Assay. C2C12 cells were transfected with the indicated plasmids for 24 h. After that, treatment with DMSO as vehicle or Imatinib (10 μM) was performed for 18 h. Finally, whole-cell lysates were collected, and luciferase and β-galactosidase activities were determined. **(B)** c-Abl inhibition decreases Pax7 transcriptional activity. Cells treated with Imatinib (mean: 0.305 ± 0.04) exhibits a significant decrease in Pax7 transcriptional activity in comparison with cells treated with vehicle (mean 1.00 ± 0.0), (*n* = 4, *P-value < 0.05, Wilcoxon signed-rank test). **(C)** WB from reporter assay cells. We observed a decrease of c-Abl phosphorylation in cells treated with Imatinib corroborating the effectiveness of the treatment.

Considering that c-Abl could regulate Pax7 via direct phosphorylation and that Imatinib affects Pax7 expression, we explored the possibility that c-Abl regulates Pax7 activity as a transcription factor. For this, we performed a luciferase reporter assay using the Pax3/7 specific reporter gene 6xPRS9-Luc (Olguin et al., 2007). C2C12 cells, maintained in proliferation culture conditions, were co-transfected with the 6xPRS9-Luc and CMV-LacZ (transfection control) vectors, plus or minus myc-Pax7 or Pax7-FKHR (positive control) expression vectors. After 24 h, transfected cells were treated with vehicle or Imatinib for 18 h prior to lysis (Figure 5A). We observed a significant decrease (>2-fold) in Pax7-induced reporter activity upon c-Abl inhibition (Figure 5B). As expected, Pax7-FKHR transfected cells showed a transcriptional activity ∼40-fold higher than control and >100-fold higher than cells treated with Imatinib. Myc-Pax7 expression was corroborated by Western blot (Figure 5C). Moreover, we observed decreased c-Abl phosphorylation, as expected upon Imatinib treatment (Figure 5C). In the context of our previous results, these findings are consistent with the idea that c-Abl regulates Pax7 activity and expression.

### Deletion of c-Abl Impairs Primary Myoblasts Function *ex vivo*

To explore the functional consequences of c-Abl dependent regulation of Pax7, we used a c-Abl loss of function approach *in vivo*. Specifically, we generated the *Abl1*^flox/flox^-Pax7^creERT2^ mouse model, which allows SC-specific c-Abl deletion, upon tamoxifen (TMX) administration (see section “Materials and Methods”).

**FIGURE 6:**
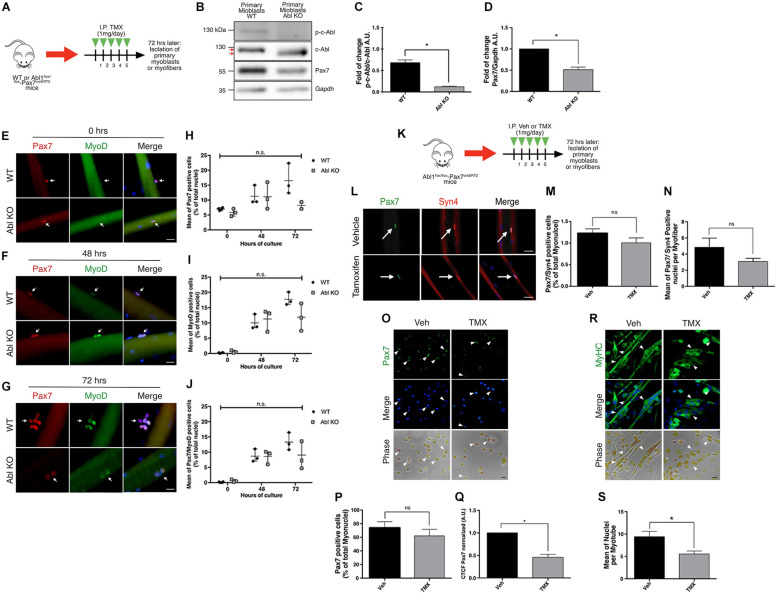
Specific deletion of c-Abl in SCs impairs myogenesis. **(A)** *In vivo* recombination protocol. *Abl1*^WT/WT^-Pax7^creERT2^ (WT) or *Abl1*^flox/flox^-Pax7^creERT2^ (Abl KO) mice were injected intraperitoneally (I.P.) with Tamoxifen (TMX, 1 mg daily) for 5 consecutive days. Then, after 72 h, myofibers or primary myoblasts were isolated. **(B)** Decreased expression of phospho-tyr412 c-Abl (p-c-Abl) and Pax7 in Abl KO myoblasts. Primary myoblasts were maintained in PM for 48 h after isolation. Then, WB was performed using indicated antibodies. **(C)** Quantification of normalized p-c-Abl protein levels from **(B)**. A.U., arbitrary units (*n* = 3, *P-value < 0.05, Wilcoxon signed-rank test). **(D)** Quantification of normalized Pax7 protein levels from **(B)**. A.U., arbitrary units (*n* = 4, *P-value < 0.05, Wilcoxon signed-rank test). **(E–G)** Expression of Pax7 and MyoD in fiber associated myoblasts from WT and Abl KO mice, at 0, 48, and 72 h of culture. Myofibers were isolated, and IF against Pax7 (red) and MyoD (green) was performed. Nuclei were stained with Hoechst 33342 (blue). Arrows indicate satellite cells associated with the myofiber. Scale bar: 10 μm. Plots **(H–J)** show the percentage of Pax7, MyoD and Pax7/MyoD double positive cells per total nuclei, respectively (*n* = 3, *P*-value > 0.05, Multiple *t*-Student test, ns, not significant). **(K)** *In vivo* recombination protocol for following analyses. *Abl1*^flox/flox^-Pax7^creERT2^ mice were injected intraperitoneally (I.P.) with vehicle (Veh) or Tamoxifen (TMX, 1 mg daily) for five consecutive days. Then, after 72 h, myofibers or primary myoblasts were isolated. **(L)** Percentage of Pax7 positive cells do not change in recently isolated myofibers from vehicle or Tamoxifen treated mice. Myofibers were isolated, and IF against Pax7 (green) and Syndecan-4 (Syn4, red) was performed. Nuclei were stained with Hoechst 33342 (blue). Arrows indicate satellite cells associated with the myofiber. Scale bar: 10 μm. **(M)** Quantification of Pax7/Syn4 positive cells from **(L)** (*n* = 4). Veh mean: 1.22 ± 0.10%; TMX mean: 1.008 ± 0.11% (*P*-value > 0.05, Mann–Whitney test, ns, not significant). **(N)** Quantification of Pax7/Syn4 positive cells per myofiber from **(L)** (*n* = 4). (*P*-value > 0.05, Mann–Whitney test, ns, not significant). **(O)** IF of proliferative primary myoblasts from mice treated with vehicle or Tamoxifen, using anti Pax7 (green) and nuclei were stained with Hoechst 33342 (blue). Scale bar: 10 μm. **(P,Q)** Graphs show quantification of Pax7 positive nuclei and Pax7 Corrected Total Cell Fluorescence (CTCF) from **(O)**. (*n* = 4, *P-value < 0.05, Wilcoxon signed-rank test). A.U., arbitrary units. **(R)** IF of differentiated (7 days) primary myoblasts from mice treated with vehicle or Tamoxifen, using anti MyHC (green) and nuclei were stained with Hoechst 33342 (blue). Arrowheads show representative myotubes. Scale bar: 10 μm. **(S)** Quantification of nuclei per myotube from **(R)** (Veh *n* = 4 and TMX *n* = 3, *P-value < 0.05, Mann–Whitney test).

Recombination was induced by five daily TMX injections as described previously (Eon et al., 2008; Reinert et al., 2012). After a resting period of 72 h, hind limb muscles were dissected in order to isolate primary myoblasts or SCs associated with single myofibers (Figures 6A,K). First, we tested the effects of c-Abl deletion in primary myoblasts maintained in proliferation culture conditions for 48 h after isolation. Western blot analyses revealed the expression of a lower molecular weight band reactive to the anti-c-Abl antibody (Figure 6B). As described by Moresco et al. (2005), a similar protein band can be detected with variable expression levels in a tissue-specific manner. In this transgenic mouse model (same used in our study), Cre-mediated recombination results in the deletion of exon 5 of the *abl1* gene, affecting the kinase domain. As expected, the same group described that this recombinant c-Abl has no kinase activity. We confirmed this by Western blot (Figures 6B,C) and by IF (Supplementary Figure 6). Noteworthy, detection of the c-Abl-kinase-null protein varied when using different anti-c-Abl antibodies commercially available (Supplementary Figure 6), which may partially explain differences in expression levels among different tissues and cell types. Consistent with our previous results, myoblasts expressing the cAbl-kinase-null protein (Abl KO) exhibited significantly lower levels of Pax7 protein compared to controls (Figure 6D). IF analyses of SCs associated with isolated myofibers indicate no significant differences in the expression of MyoD, from 0 to 72 h (Figures 6E–J). Interestingly, Pax7 expression appears to decrease at 72 h in Abl KO SCs, however, differences did not reach statistical significance due to the high variability in the numbers of cells associated with myofibers in each case (Figures 6G,H). Next, we analyzed the number of SCs associated with myofibers by IF, immediately after isolation. Using Pax7 and Syndecan-4 as independent SC markers, we detected no significant changes in the total number of SC upon TMX administration compared to the vehicle (Figures 6L–N). The reduction of Pax7 expression was corroborated by IF, in primary myoblasts maintained in proliferation culture conditions (Figures 6O–Q). Moreover, Abl KO myoblasts exhibited a reduced differentiation capacity, evaluated by MyHC expression and by the formation of multinucleated myotubes with reduced nuclei number, compared to control myotubes (Figures 6R,S). Together, these results are consistent with the effect of c-Abl inhibition on C2C12 differentiation and suggest that c-Abl activity regulates Pax7 expression and myogenic potential in SCs.

**FIGURE 7:**
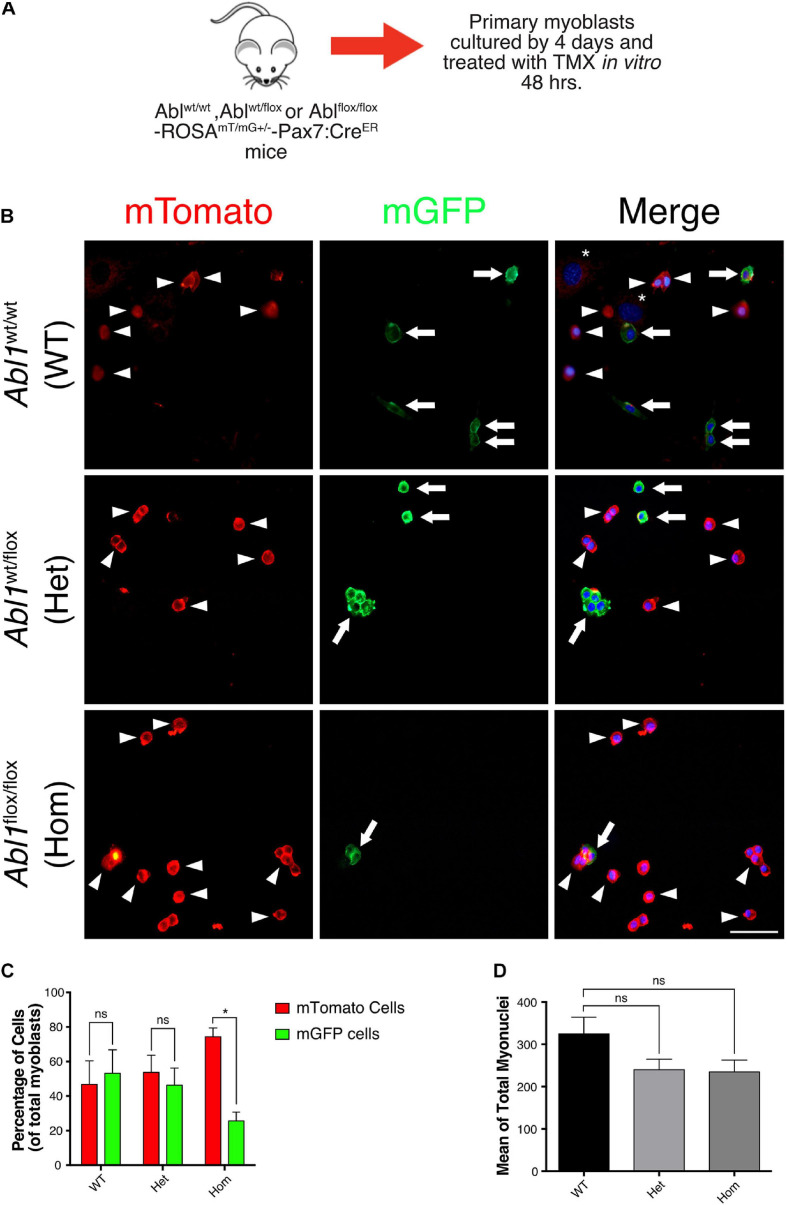
GFP positive primary myoblasts population decrease in c-Abl Knockout cells recombined *in vitro*. **(A)** Protocol to induce *Abl1* recombination *in vitro*. Primary myoblasts were isolated from *Abl1*^WT/WT^ (WT), *Abl1*^wt/flox^ (Het), or *Abl1*^flox/flox^ (Hom) ROSA^mT/mG^-Pax7^creERT2^ mice and maintained 4 days in PM. During this time, cells were treated with vehicle or Tamoxifen (TMX, 10 μM) for 48 h. After that, cells were fixed, and direct fluorescence was detected. Nuclei were stained with Hoechst 33342 (blue). **(B)** Arrowheads show mTomato positive cells and arrows show mGFP positive cells. The asterisk shows non-myogenic cells. Scale bar: 50 μm. **(C)** Quantification of the percentage of mTomato positive and mGFP positive myoblasts from **(B)** (*n* = 3, *P-value < 0.05, multiple *t*-Student test, ns, not significant). **(D)** Total number of myoblasts do not change in different mice. Mean of total myoblasts was determined for every mouse from **(B)**. WT mean: 324.5 ± 39.50; Het mean: 239.7 ± 25.06; Hom mean: 234.7 ± 28.30 (*n* = 3, *P*-value > 0.05, ANOVA test, ns, not significant).

To follow the fate of Abl KO myoblasts in culture, we used a ROSA^mT/mG^ reporter mouse in the *Abl1*^flox/flox^-Pax7^creERT2^ genetic background, allowing for lineage tracing *in vivo* and *in vitro* (see section “Materials and Methods”). To avoid differences in the starting number of cells, primary myoblasts were isolated from uninjured muscles, and treated with TMX for 48 h to induce recombination *in vitro* (Figure 7A). After additional 96 h, cells were fixed, and the expression of mTomato (non-recombined cells) or mGFP (recombined cells) was analyzed by direct fluorescence (Figure 7B). We observed a similar proportion of cells expressing mTomato and mGFP in myoblast cultures from WT and *Abl1*^*wt/flox*^ mice (Figure 7C). However, we detected a significant difference in mTomato (74.38 ± 5.06%) versus mGFP (25.61 ± 5.06%) expressing cell populations in *Abl1*^flox/flox^ myoblast cultures. Noteworthy, the average total number of nuclei was not significantly different among conditions (Figure 7D), suggesting that proliferation and/or survival were specifically affected in mGFP(+) cells (i.e., Abl KO myoblasts).

### Muscle Regeneration Is Disturbed in SC-Abl KO Mice

**FIGURE 8:**
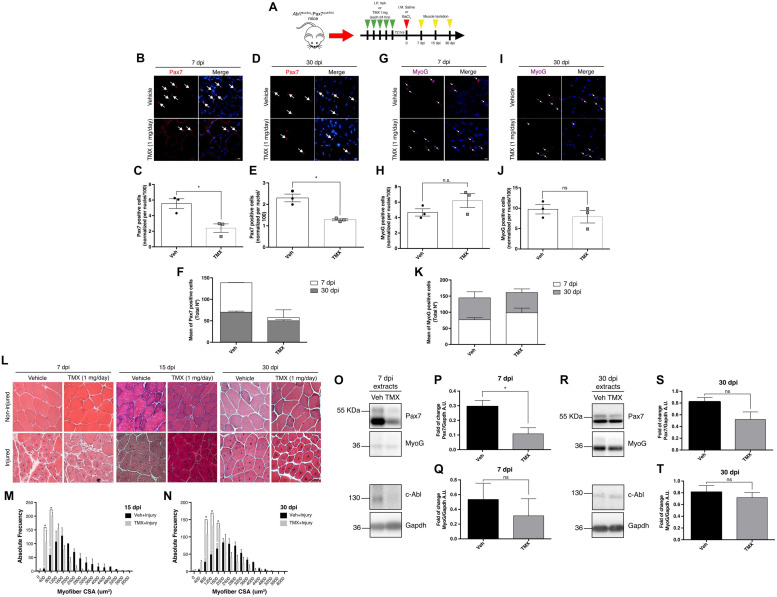
Specific deletion of c-Abl in SCs impairs muscle regeneration. **(A)** Protocol for muscle regeneration in *Abl1*^flox/flox^-Pax7^creERT2^. Mice were injected intraperitoneally (I.P.) with vehicle (Veh) or Tamoxifen (TMX, 1 mg daily) for five consecutive days. Then, after 72 h, muscle injury was induced by intramuscular (I.M.) injection of BaCl_2_ in the tibialis anterior (TA) muscle. Contralateral muscles were injected with saline solution as control. Next, muscles were isolated at 7-, 15-, and 30-days post-injury (dpi). **(B,D)** Pax7 positive cells decrease in SC-Abl KO mice during muscle regeneration. IF against Pax7 (red) was performed in 7 dpi **(B)** and 30 dpi **(D)** muscle sections. Nuclei were stained with Hoechst 33342 (blue). Arrows show Pax7 positive cells. Scale bar: 10 μm. Plots **(C,E)** show quantification for Pax7 positive cells normalized per 100 nuclei for 7 and 30 dpi respectively (*n* = 3, *P-value < 0.05, Mann–Whitney test). **(F)** Graph shows the mean of total Pax7 positive cells by mouse. **(G,I)** Myogenin (MyoG) positive cells in SC-Abl KO mice during muscle regeneration. IF against MyoG (magenta) was performed in 7 dpi **(G)** and 30 dpi **(I)** muscle sections. Nuclei were stained with Hoechst 33342 (blue). Arrows show MyoG positive cells. Scale bar: 10 μm. Plots **(H,J)** show quantification for MyoG positive cells normalized per 100 nuclei for 7 and 30 dpi respectively (*n* = 3, *P*-value > 0.05, Mann–Whitney test, ns: not significant). **(K)** Graph shows the mean of total MyoG positive cells by mouse. **(L)** Decrease in cross-sectional area (CSA) of regenerative myofibers from c-Abl KO mice. Sections from TA muscles were stained with Hematoxylin-Eosin stain. Upper panels: non-injured contralateral muscles; Lower panels: injured TA. Scale bar: 50 μm. **(M)** Distribution of CSA of regenerative myofibers at 15 dpi. **(N)** Distribution of CSA of regenerative myofibers at 30 dpi (*n* = 3, *P-value < 0.05, Two-way ANOVA test). **(O,R)** Pax7 protein levels decrease during muscle regeneration in c-Abl KO mice. 7 and 30 dpi TA were lysed, and WB using anti-Pax7, anti-Myogenin (MyoG), or anti-c-Abl was performed. Gapdh was used to normalize protein levels. **(P,Q)** Quantification of normalized Pax7 and MyoG protein levels from 7 dpi extracts **(O)**. A.U., arbitrary units (*n* = 3, *P-value < 0.05, Mann–Whitney test, ns: not significant). **(S,T)** Quantification of normalized Pax7 and MyoG protein levels from 30 dpi extracts **(R)**. A.U., arbitrary units (*n* = 3, *P*-value > 0.05, Mann–Whitney test, ns, not significant).

Based on our previous results, we hypothesized that SC function would be altered *in vivo* upon *Abl1* deletion, affecting the outcome of muscle regeneration. To test this concept, muscle damage was induced by intramuscular injection (TA muscles) of 1.2% barium chloride (BaCl_2_) (Cornelison et al., 2004) in *Abl1*^flox/flox^-Pax7^creERT2^ mice, previously treated with TMX (SC-Abl KO) or vehicle as described. Contralateral muscles were injected with saline solution (NaCl 0.9%) as internal control. TA muscles were collected at different days post injury (dpi) for downstream analyses (Figure 8A). Correlating with previous findings, IF analyses showed a significant reduction in the number of Pax7(+) cells per field at 7 and 30 dpi in SC-Abl KO mice, compared to injured muscles from vehicle-treated animals (Figures 8B–E). The number of Pax7(+) cells are expected to increase after activation (during early phases of regeneration), and later decline reaching the numbers observed during quiescence. This change in the Pax7(+) population is observed in injured muscles treated with vehicle, but is absent in SC-Abl KO muscles (Figure 8F), suggesting an impaired expansion of the progenitor population. This idea is supported in part by the reduction in the number of MyoD(+) cells in SC-Abl KO muscles at 7 dpi, compared to control (Supplementary Figure 7). No significant changes were observed in the number of Myogenin(+) cells between SC-Abl KO and control muscles, both at early (7 dpi) or late (30 dpi) regeneration (Figures 8G–K).

Histological analysis using H&E staining revealed the expected changes in tissue architecture during regeneration (Figure 8L) and an overall reduction in fiber size in SC-Abl KO animals compared to control, quantified at 15 and 30 dpi (Figures 8M,N). Myofiber cross-sectional area (CSA) measurements revealed a significant difference in the distribution of fiber size, consistent with the accumulation of small caliber myofibers in SC-Abl KO regenerating muscles.

To test if proliferation was affected in SC-Abl KO mice, we performed IF analysis of ki67 expression in 7 dpi sections. Interestingly, although not significant, we observed a decrease in the percentage of ki67 positive nuclei in TMX treated animals compared to vehicle (Supplementary Figure 8). This observation agrees with our previous results in primary myoblasts and suggests that could be a proliferation defect in c-Abl KO myoblasts.

Finally, we performed Western blots to analyze Pax7 protein levels from whole muscle extracts at 7 and 30 dpi. In accordance with IF analyses, we observed a significant decrease in Pax7 protein levels at 7 dpi in SC-Abl KO muscles (Figures 8O,P). Although not statistically significant, a decrease in Myogenin protein levels were also observed in regenerating SC-Abl KO mice (Figures 8O,Q). As expected from the previous analysis, decreased Pax7 and Myogenin protein levels still are observed at 30 dpi in SC-Abl KO muscles, however, these differences are not statistically different from control values (Figures 8R–T).

To further confirm c-Abl-Pax7 interaction *in vivo*, we performed co-IP from whole muscle extracts obtained at 30 dpi (see section “Materials and Methods”). Surprisingly, we detected that c-Abl co-immunoprecipitate with Pax7 in samples from injured and non-injured muscles, but c-Abl signal is lower for TMX-treated animals (Supplementary Figure 9). Noteworthy, we did not detect the c-Abl-kinase-null protein from whole-muscle extracts, which is consistent with the tissue-specific expression variability reported previously (Moresco et al., 2005).

Together, our *in vivo* studies indicate that c-Abl deletion impairs satellite cell function during regeneration. Consistent with our previous data, these results also suggest that c-Abl could regulate myogenesis via the control of Pax7 function and expression.

## Discussion

The present study uncovers a new target for c-Abl activity during myogenesis. Our results suggest that c-Abl controls Pax7 expression and activity in adult muscle progenitors, potentially by directly phosphorylating Pax7 protein. Importantly, c-Abl loss-of-function impairs muscle differentiation without a significant change in MyoD or Myogenin expression, indicating that c-Abl could regulate myogenesis by promoting Pax7 expression and function.

By using C2C12 cells, primary myoblasts and a muscle injury-and-regeneration model in mice, we provide a more detailed characterization of c-Abl expression pattern during muscle differentiation. Our findings indicate that: (i) levels of c-Abl phosphorylated at tyrosine 412 increase in activated muscle progenitors during early muscle regeneration; (ii) levels of phospho-c-Abl decreased in differentiated cells; and (iii) c-Abl is localized to the cytoplasm in Pax7(–) cells.

It is known that c-Abl shuttles between nucleus and cytoplasm during differentiation, and also that c-Abl activity is higher in proliferative versus differentiated cells (di Bari et al., 2006; Bae et al., 2009). Our observations highlight a correlation between c-Abl activity and localization with the myogenic progression of activated muscle progenitors. Specifically, our results show that c-Abl phosphorylated at tyrosine 412 is present in myogenic cells during the first 3 days of muscle regeneration but not after 7 days neither in non-injured muscle. We also determined that c-Abl inhibition impairs differentiation of C2C12 cells, decreasing the number of multinucleated cells expressing MyHC. These results are similar to those obtained by Bae et al. (2009) in which using a c-Abl siRNA in C2C12 cells, they noted a decrease in myotube formation. In our study, we observed a reduction in the total number in Myogenin expressing cells upon c-Abl inhibition, however the number of MyoD/Myogenin positive cells remained unchanged. These observations support the idea that c-Abl could regulate myogenesis upstream the induction of Myogenin.

Previously, it has been described that c-Abl regulates MyoD activity by phosphorylation, resulting in a decrease in transcriptional activity and myogenesis arrest in conditions where cells suffer DNA damage (Puri et al., 2002; Innocenzi et al., 2011; Simonatto et al., 2013). These studies showed that c-Abl can regulate MyoD activity, however the effect on MyoD protein levels were not explored. Since MyoD binding to DNA prevents its degradation (Abu Hatoum et al., 1998), we hypothesized that c-Abl-dependent regulation of MyoD activity could directly or indirectly affect MyoD protein levels. Therefore, we decided to analyze the expression of myogenic transcription factors in cells treated with a c-Abl inhibitor. Contrary to our first idea, MyoD protein levels did not significantly decrease when c-Abl was inhibited. Unexpectedly, we observed a consistent decrease in Pax7 detection in IF studies. This effect was detected as a decrease in the levels of Pax7 signal per cell, rather than a decrease in the number of Pax7(+) cells; later corroborated by Western blot analysis. Conversely, using the c-Abl activator DPH (Yang et al., 2011), we observed a significant increase in Pax7 protein levels, both by IF and Western blot. Noteworthy, we observed a reduction in Pax7 mRNA levels that preceded decreased protein levels, suggesting a role for c-Abl in the regulation of *pax7* transcription. Previously, it has been shown that TNF/p38α promotes PRC2 recruitment to repress Pax7 gene expression in differentiating myoblasts (Palacios et al., 2010). This could be related to c-Abl-dependent activation of p38α/β MAPK during differentiation (Bae et al., 2009), but does not explain diminished Pax7 expression upon c-Abl inhibition. On the other hand, it has been described that c-Abl phosphorylates Emerin (Tifft et al., 2009), an inner nuclear membrane protein involved in several processes including Pax7 loci localization at nuclear lamina during differentiation, which leads to its transcriptional repression (Demmerle et al., 2013). Future studies could determine if c-Abl inhibition affects Pax7 expression by an Emerin-dependent mechanism.

Exploring the functional interaction between c-Abl and Pax7, we show that both proteins can be co-immunoprecipitated from cell and whole muscle extracts. Moreover, we provide evidence for the first time that Pax7 is phosphorylated by c-Abl on tyrosine residue(s) using an *in vitro* phosphorylation assay. Preliminary bioinformatic analysis of motifs in Pax7 protein performed on Group-based Prediction System (GPS) 5.0 (Wang et al., 2020) and Scansite 4.0 (Obenauer et al., 2003) suggests at least one residue with a high probability of being phosphorylated by c-Abl: Tyr440 (not shown). We and others described that Pax7 phosphorylation on serine residues is key to avoid its degradation (Dick et al., 2015; González et al., 2016). In the current study, we could not detect recovery of Pax7 levels when C2C12 cells are treated with Imatinib and the proteasome inhibitor MG132 (Supplementary Figures 10A,B). In addition, we did not observe an increase of Pax7 ubiquitination (determined by BiFC) in cells treated with Imatinib, compared to vehicle (Supplementary Figure 10D). These results do not exclude other degradation pathways, like caspase-mediated cleavage of Pax7 (Olguín, 2011; Dick et al., 2015).

A potential outcome of c-Abl-dependent Pax7 phosphorylation relates to its transcriptional activity. Indeed, reporter-gene assays indicate that c-Abl inhibition decreases Pax7-dependent activation of the 6xPRS9-luc reporter. Previously, we described that CK2-dependent phosphorylation of Pax7 did not affect its transcriptional activity (González et al., 2016), highlighting a distinct role of c-Abl regulation over Pax7. In addition, it has been shown that other post-translational modifications of Pax7, like methylation and SUMOylation, are required for the induction of its target genes (Kawabe et al., 2012; Luan et al., 2013). This interaction between different post-translational modifications over Pax7 function grants further investigation.

Lee et al. (2017) described no relevant effects of c-Abl deletion on muscle fiber development in embryonic stages. However, to the best of our knowledge, there are no studies regarding c-Abl specific ablation on SCs and how this impacts muscle maintenance and regeneration. We explored this point by generating a *Abl1*^flox/flox^-Pax7^creERT2^ transgenic mouse, which allows inducible loss of c-Abl expression in SCs. SC-specific c-Abl ablation had no significant effect in the number of Pax7(+) cells associated with individual myofibers, isolated from uninjured muscles. However, when c-Abl KO myoblasts were isolated and maintained in proliferation or differentiation conditions, we observed a significant decrease in the number of Pax7(+) cells and a significant differentiation impairment. Both observations are remarkably similar to the effect of c-Abl inhibition *in vitro*.

Using lineage tracing of recombined cells, we detected that those cultures in which c-Abl was totally deleted exhibits a diminished recombinant myoblast population in comparison with those in which the kinase remains total or partially expressed. This result suggests that c-Abl is required for proliferation and/or survival of myoblasts. Several studies have shown that c-Abl participates in cell cycle and apoptosis regulation in different contexts (reviewed in Wang, 1993, 2014). However, it is unclear whether these mechanisms are operating in myoblasts. On the other hand, our observation could be due to decreased Pax7 levels, since deletion of Pax7 led to cell cycle defects and loss of SCs pool caused by cell death (Relaix et al., 2006). Further proliferation and viability analyses in myoblasts lacking c-Abl could reveal more details about this question.

Importantly, we detected that *in vivo* muscle regeneration is significantly impaired upon SC-specific c-Abl deletion, resulting in a lower regenerative potential and a lower myofiber CSA in comparison with control mice. Furthermore, this reduction in regenerative capacity is accompanied by lower Pax7 protein levels. In fact, this protein is not only responsible for the myogenic specification of SCs (Seale et al., 2000; Blake and Ziman, 2014), but also has a fundamental role in muscle regeneration. Conditional deletion of Pax7 in SCs from adult mice results in a reduction of muscle weight, number and caliber of regenerated myofibers upon cardiotoxin induced damage (von Maltzahn et al., 2013). These observations are in agreement with our results, suggesting that c-Abl ablation impairs muscle regeneration, at least in part, by decreasing Pax7 levels. Additionally, c-Abl is involved in several critical processes for differentiation of SCs, such as the activation of MEK5/ERK5 MAPK and p38α/β MAPK cascades and promoting cadherin-mediated cell–cell adhesion of myoblasts (Bae et al., 2009; Lu and Krauss, 2010; Chen et al., 2017). Altogether, these observations suggest that c-Abl is required in both, early and later stages of myogenesis, regulating more than one molecular pathway depending on its subcellular localization.

Other groups have shown that c-Abl inhibitors, including Imatinib and Nilotinib, also affect myogenic differentiation and improve the dystrophic phenotype in *mdx* mice (Ito et al., 2013; Lemos et al., 2015; Contreras et al., 2018). The underlying molecular pathways, however, were associated with the regulation of p38/MAPK and inhibition of profibrotic effects of TGF-β. On the other hand, a recent study reports muscle toxicity induced by the use of tyrosine kinase inhibitors, as Imatinib, in patients with chronic myeloid leukemia (Janssen et al., 2019). The authors relate this toxicity to mitochondrial dysfunction; in light of our results, it is possible that disruption of SC function adds to decreased muscle health in these individuals.

Here we show a different type of myogenic regulation mediated by c-Abl regarding Pax7 activity and its expression control, placing tyrosine phosphorylation signaling as a novel player in Pax7 regulation and as an attractive target to manipulate myoblasts decisions. However, the determination of specific tyrosine residue(s) phosphorylated in Pax7, or whether c-Abl interaction with Pax7 depends on other proteins which regulate its function remains unclear. Ongoing studies in our laboratory seek to uncover more details about the mechanism regarding this new pathway.

## Data Availability Statement

The original contributions presented in the study are included in the article/Supplementary Material, further inquiries can be directed to the corresponding author/s.

## Ethics Statement

The animal study was reviewed and approved by the Pontificia Universidad Católica de Chile Bioethics and Biosecurity Committee (protocol ID 160929002).

## Author Contributions

HO and FM conceived and designed the experiments. FM performed most of the experiments, processed the experimental data, performed the quantification analyses, and designed the figures. NG carried out the immunoprecipitation, phosphorylation, BiFC assays, and data analysis. NB performed the qRT-PCR and reporter assays. MR performed the Western blot analyses for c-Abl, p-c-Abl, and c-Abl-kinase-null proteins from myoblast cultures, and Imatinib two-dose treatment experiment. AA provided the *Abl1*^*flox*^ animal model. AG-M helped to generate transgenic lines. FM, HO, and NG wrote and prepared the manuscript. All the authors discussed the results giving critical feedback.

## Conflict of Interest

The authors declare that the research was conducted in the absence of any commercial or financial relationships that could be construed as a potential conflict of interest.
