# Supplementary material for: c-Abl Kinase Is Required for Satellite Cell Function Through Pax7 Regulation
Source: Front Cell Dev Biol. 2021 Mar 11;9:606403. doi: 10.3389/fcell.2021.606403 (PMC7990767; doi:10.3389/fcell.2021.606403)
Supplement: Supplementary file 1 [file Data_Sheet_1.pdf]

Supplementary data.

Supplementary methods.

Ubiquitin-mediated bimolecular fluorescence complementation (BiFC).

General BiFC protocol was performed as described previously (Bustos et al., 2015) with minor modifications. Briefly, cells were transfected with plasmids encoding the C-terminal fragment of Venus fluorescent protein (VC155) fused to Pax7, and the N-terminal fragment of Venus (VN155-I152L) fused to Ubiquitin. VC-bFos and VN-bJun were transfected separately as BiFC-positive control. Gap43-mRFP was included as a transfection marker in all conditions. 24 hours post-transfection, cells were treated with vehicle, 10 µM Imatinib or DPH for 24 hours. All cells were treated with 25 µM MG132 (proteasome inhibitor) during the 6 hours prior to fixation with 4% PFA. Nuclei were stained with Hoechst 33342 and direct fluorescence was evaluated.

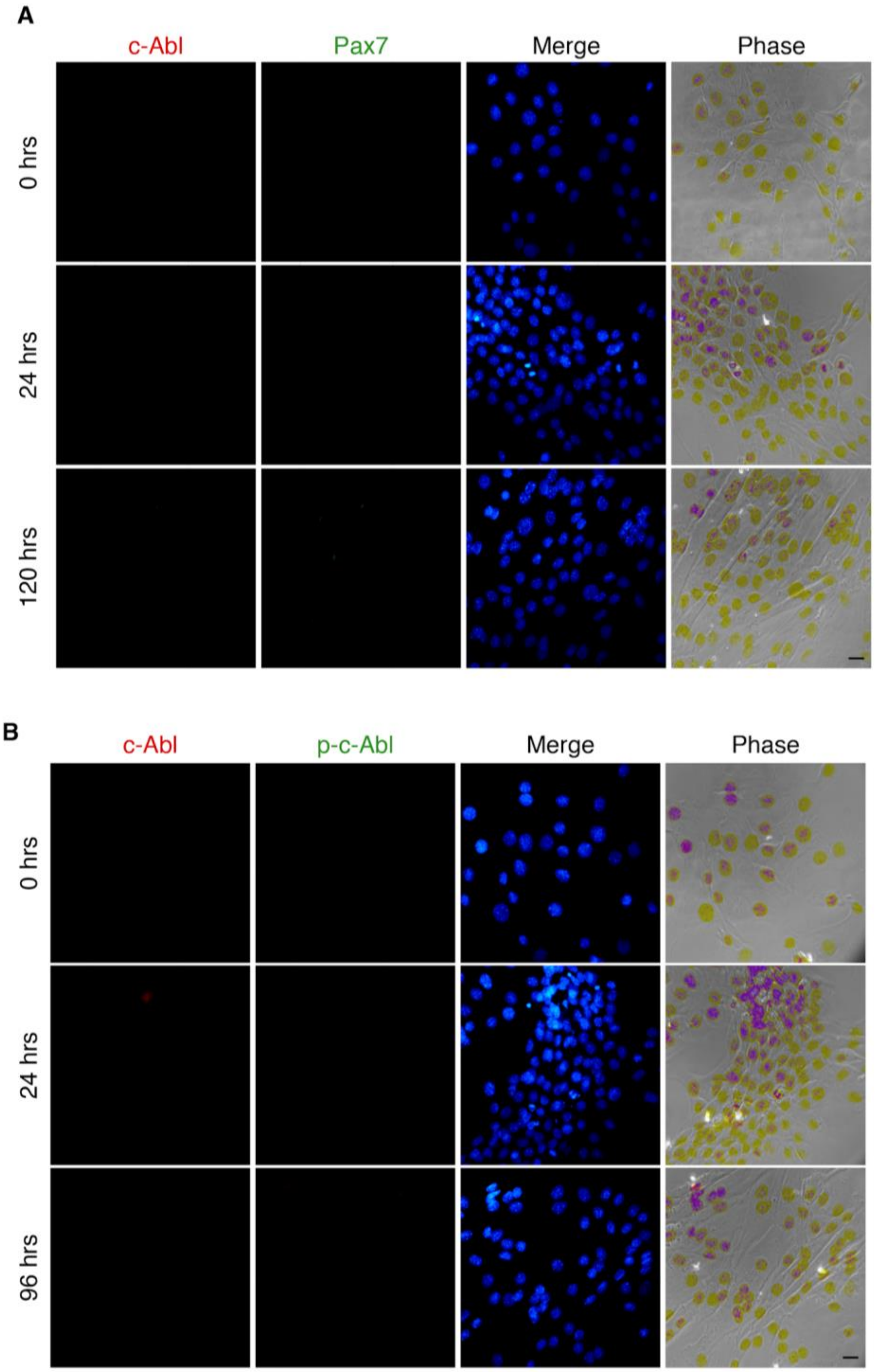

Supplementary Figure 1. Secondary antibody controls for C2C12 differentiation immunofluorescence. A) Only secondary antibodies anti-mouse IgG (green) and anti-rabbit IgG (red) were incubated with C2C12 cells at 0, 24 and 120 hours of differentiation. Nuclei were stained with Hoechst 33342 (blue). Scale bar: 10 μm. B) Only secondary antibodies anti-rabbit IgG (green) and anti-mouse IgG (red) were incubated with C2C12 cells at 0, 24 and 96 hours of differentiation. Nuclei were stained with Hoechst 33342 (blue). Scale bar: 10 μm.

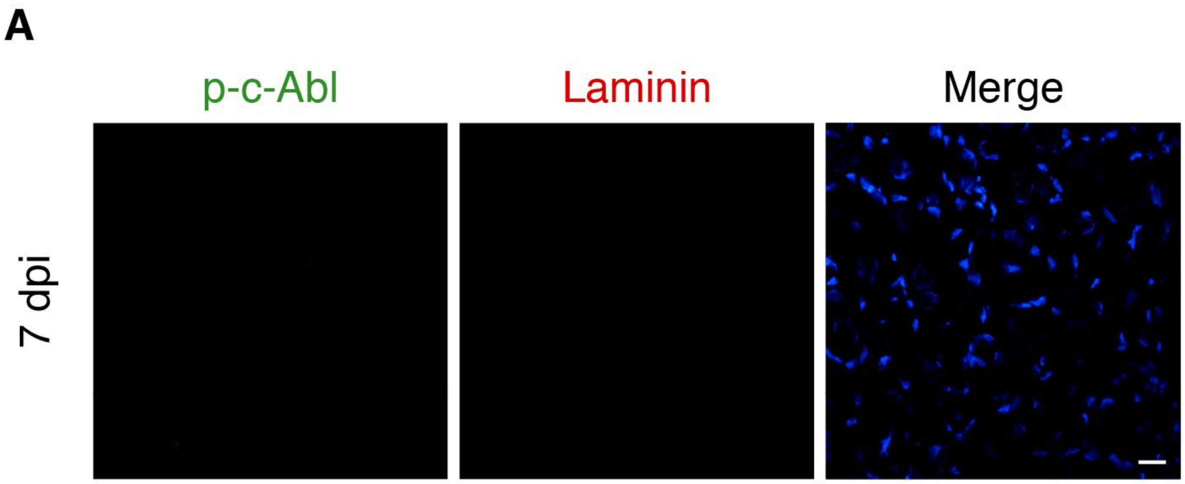

Supplementary Figure 2. Secondary antibody controls for tissue sections immunofluorescence. A) Only secondary antibodies anti-rabbit IgG (green) and anti-rat IgG (red) were incubated with tissue sections of 7 days post injury (dpi) tibialis anterior. Nuclei were stained with Hoechst 33342 (blue). Scale bar: 10 μm.

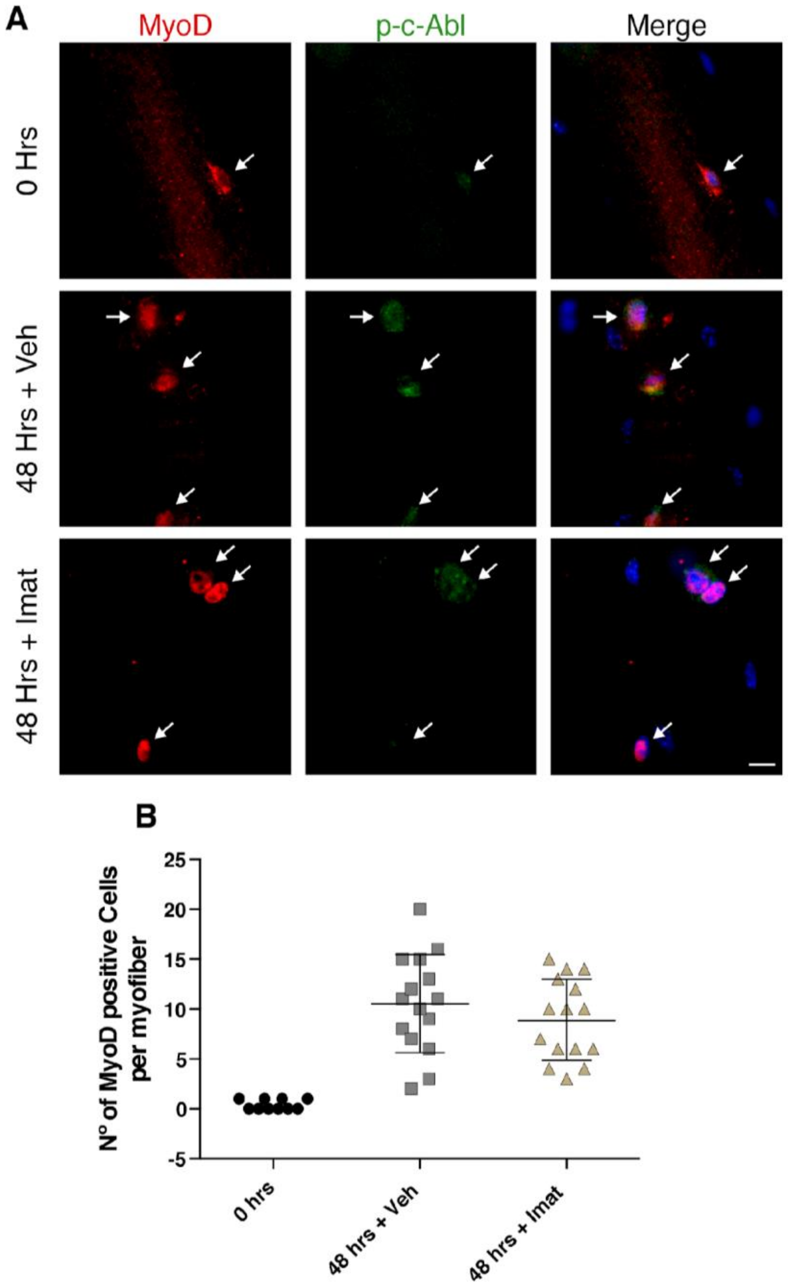

Supplementary Figure 3. Imatinib treatment does not affect the number of MyoD positive cells. A) Cultured myofibers treated with Imatinib. Myofibers were isolated, cultured for 48 hours and treated with Vehicle or Imatinib (10µM). IF against MyoD (red) and phospho-c-Abl (p-c-Abl, green) was performed. Nuclei were stained with Hoechst 33342 (blue). Arrows indicate satellite cells associated with the myofiber. Scale bar: 10 µm. B) Quantification of MyoD positive cells per myofiber from A (n=1).

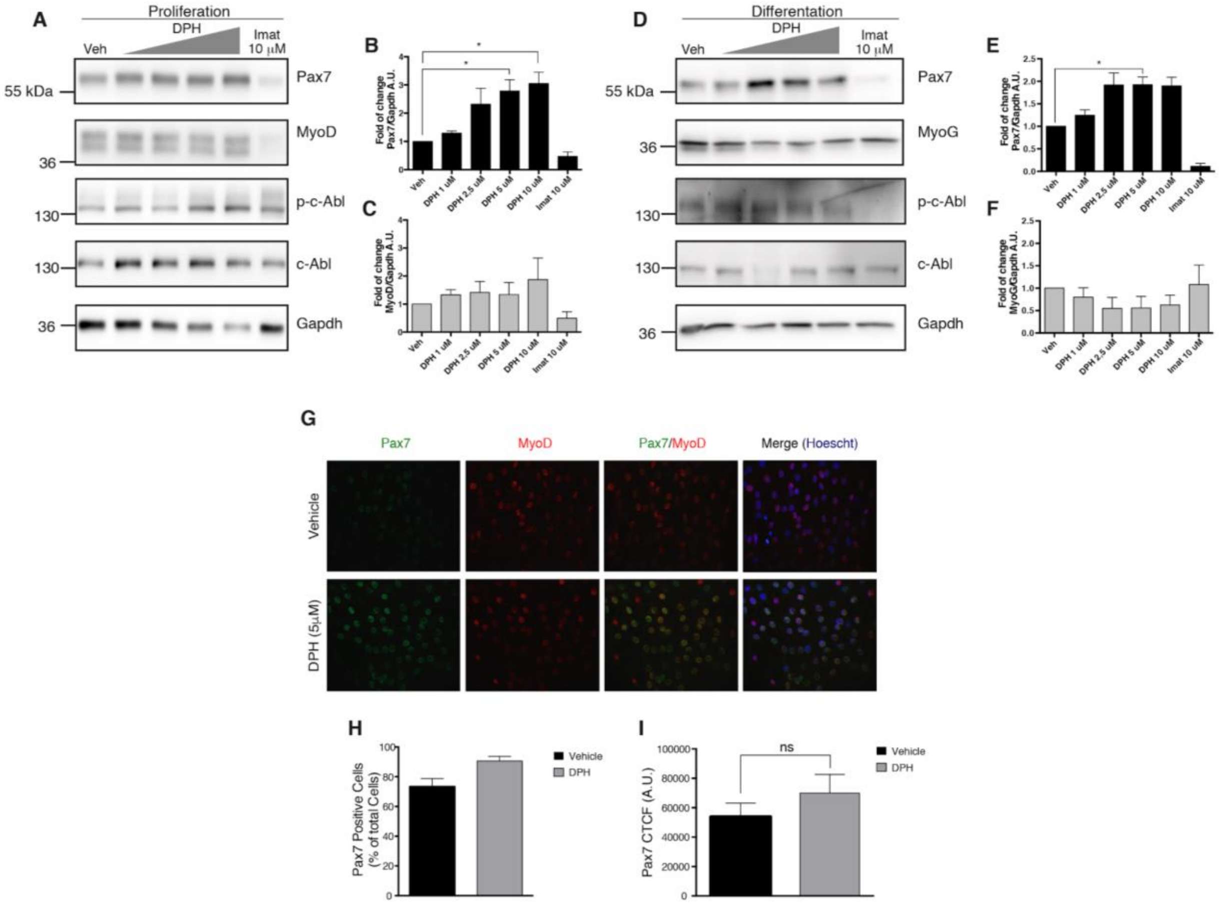

Supplementary Figure 4. c-Abl up-regulated activity increases Pax7 expression. A) y D) DPH increases Pax7 protein levels in proliferating and differentiating C2C12 cells. C2C12 cells were seeded on a 6 multiple wells plate in PM or DM, overnight. The next day, cells were treated with vehicle (Veh), DPH (0, 1, 2.5, 5 or 10 μM) or Imatinib (Imat, 10 μM) every 24 hours, during two consecutive days. After that, cells were lysed, and WB was performed using the antibodies indicated. B and E) Quantification of Pax7 levels shown in A and D (n=5, *P-value < 0.05, Mann-Whitney test). C) Quantification of MyoD protein levels shown in A (n=3). F) Quantification of Myogenin (MyoG) levels shown in D (n=3). G) DPH increases Pax7 expression in C2C12 cells *in vitro*. Cells were maintained in proliferation for 48 hours and treated as indicated. Then cells were fixed and prepared to do IF against Pax7 (green) and MyoD (red). Cell nuclei were stained with Hoechst 33342 (blue). Scale bar: 10 μm. H) Percentage of Pax7 positive cells from G. I) Corrected Total Cell Fluorescence (CTCF) determined from G. The Pax7 intensity was determined using the ImageJ software (n=3, P-value > 0.05, Mann-Whitney test, ns: not significant).

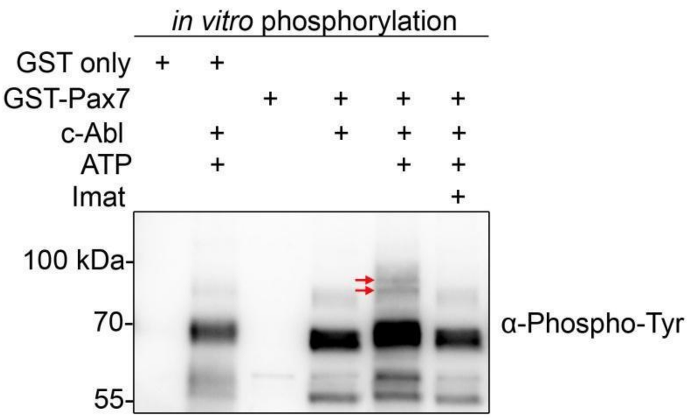

Supplementary Figure 5. Negative control of Pax7 *in vitro* phosphorylation. GST only or GST-Pax7 were incubated with c-Abl plus or minus ATP, for *in vitro* phosphorylation assay (see Materials and Methods). Red arrows show phospho-Tyr-Pax7 bands, which are absent in GST only control.

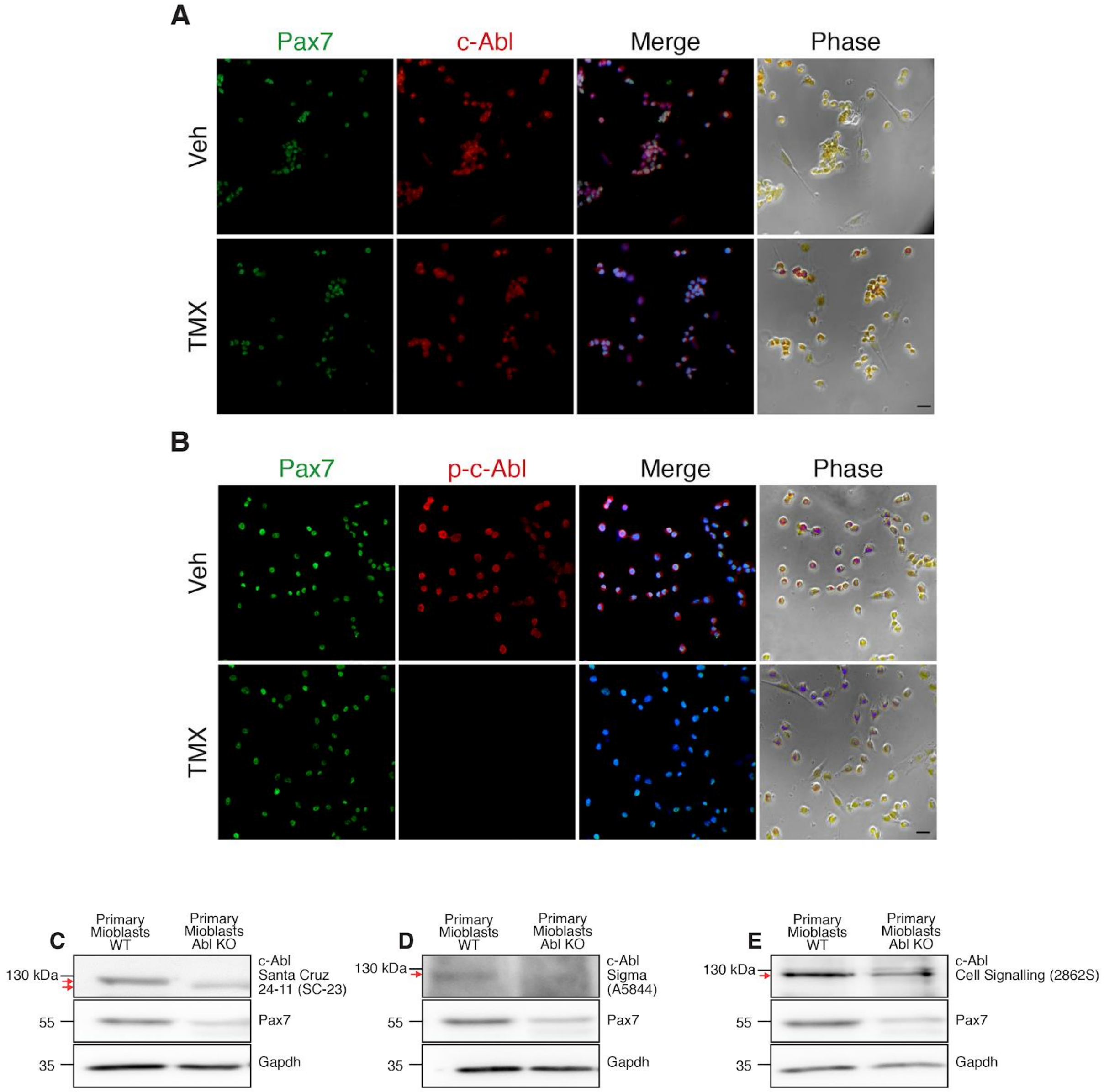

Supplementary Figure 6. Expression of inactive c-Abl in primary myoblasts from SC-Abl KO mice. A) and B) Detectable levels of c-Abl but not phospho-tyr412 c-Abl (p-c-Abl) in Abl KO myoblasts. IF against c-Abl (red) and Pax7 (green) (A) or p-c-Abl (red) and Pax7 (green) (B) was performed in myoblast cultures from vehicle or tamoxifen (TMX) treated SC-Abl KO mice. Nuclei were stained with Hoechst 33342 (blue). C), D) and E) Western blot analyses of c-Abl KO myoblasts revealed distinct bands depending on c-Abl antibody used. WB was performed with the indicated antibody in samples from myoblast cultures of *Abl1*$^{WT/WT}$-Pax7^{creERT2} (WT) or *Abl1*$^{flox/flox}$-Pax7^{creERT2} (Abl KO). Red arrows show the c-Abl band(s).

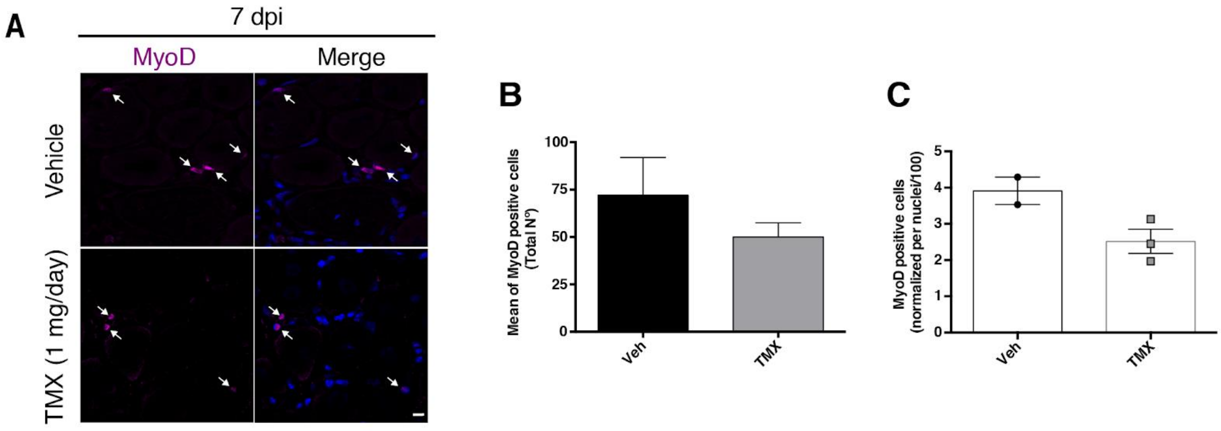

Supplementary Figure 7. Decreased MyoD expression in SC-Abl KO regenerating muscles. A) Sections of 7 dpi TA of Vehicle (Veh) or Tamoxifen (TMX) treated animals were subjected to IF against MyoD (magenta). Nuclei were stained with Hoechst 33342 (blue). B) Quantification of A for the mean of total MyoD positive cells by mouse. C) Quantification of MyoD positive cells normalized per 100 nuclei at 7 dpi (Veh n=2, TMX n=3).

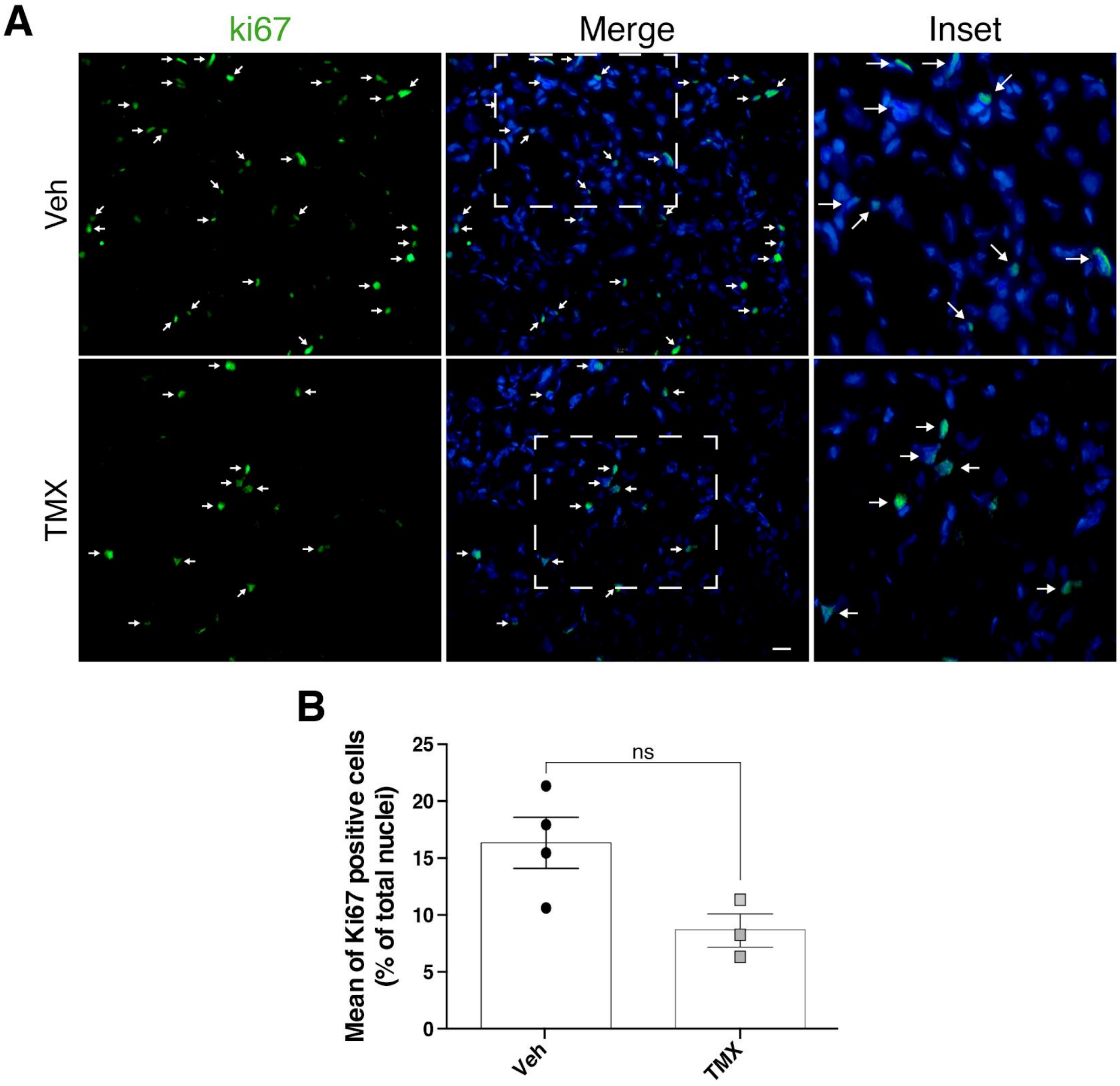

Supplementary Figure 8. Proliferation marker ki67 is decreased in SC-Abl KO regenerating muscles. A) Sections of 7 dpi TA of Vehicle (Veh) or Tamoxifen (TMX) treated animals (SC-Abl KO mice) were subjected to IF against ki67 (green). Nuclei were stained with Hoechst 33342 (blue). Arrows show ki67 positive nuclei. B) Quantification of percentage of ki67 positive nuclei per total nuclei (Veh n=4, TMX n=3, P-value > 0.05, Mann-Whitney test, ns: not significant).

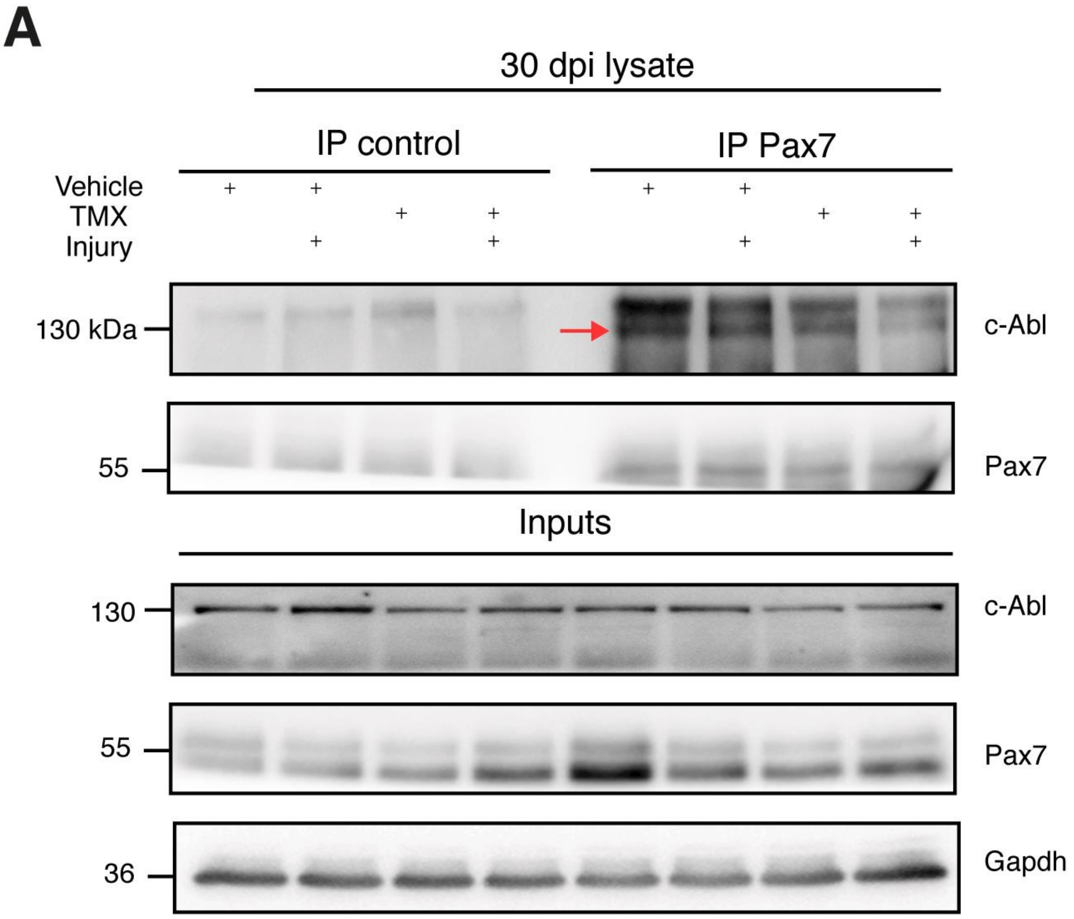

Supplementary Figure 9. c-Abl co-immunoprecipitates with Pax7 in muscle. A) 30 dpi TA were lysed, and immunoprecipitation (IP) with anti-normal mouse IgG as control or anti-Pax7 was performed, followed by WB for c-Abl and Pax7 (Upper panels). The red arrow shows the band that corresponds to c-Abl. Lower panels show inputs for c-Abl, Pax7, and Gapdh.

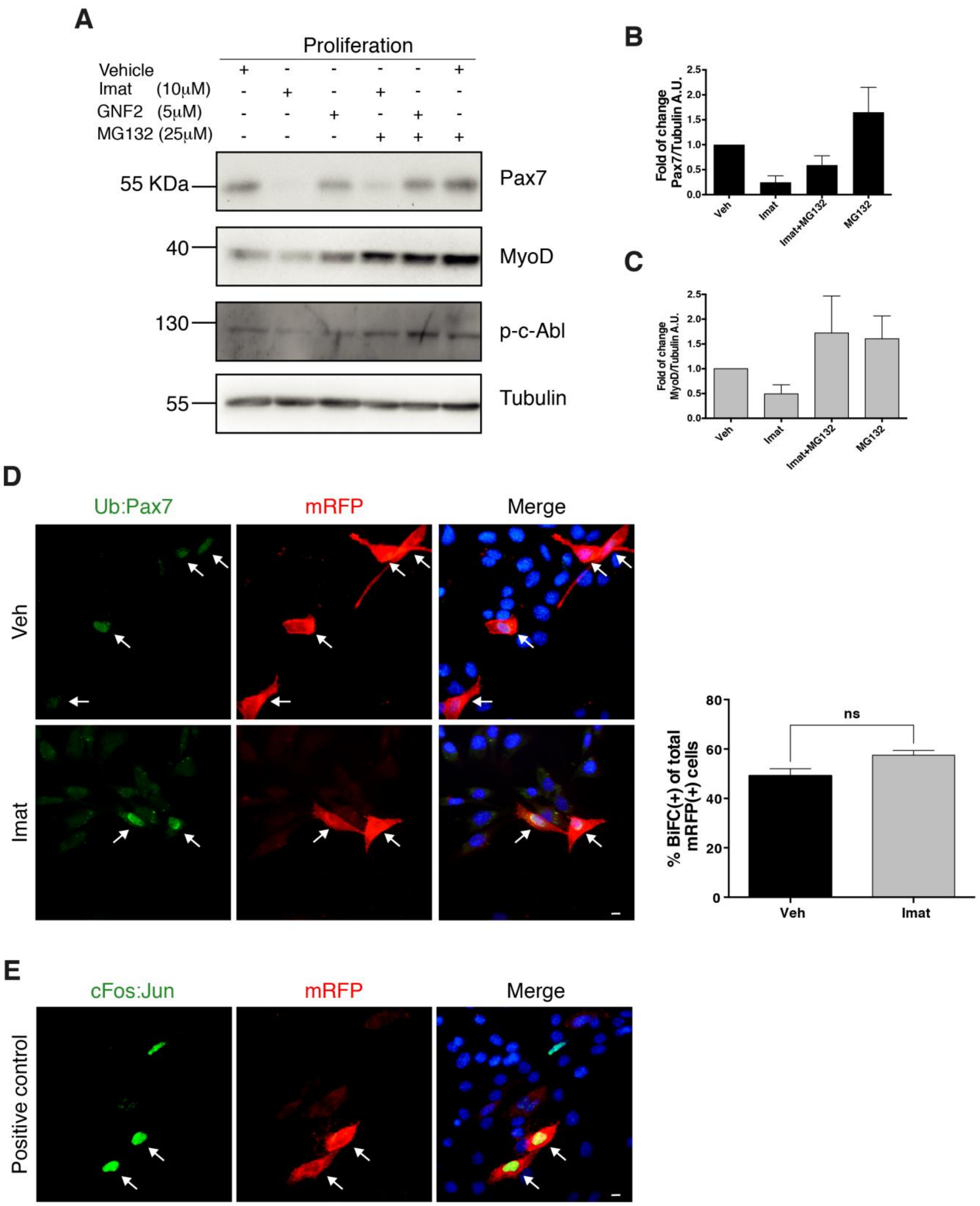

Supplementary Figure 10. UPS is not involved in Pax7 decreased levels induced by c-Abl inhibition. A) Pax7 protein levels do not recover with Ubiquitin Proteasome System (UPS) inhibition. C2C12 cells were seeded in PM or DM, overnight. The next day, cells were treated with vehicle, Imatinib (10 μM), or GNF2 (5 μM) every 24 hours, during two consecutive days. Five hours before lysis, cells were treated with proteasome inhibitor MG132 (25 μM). After that, cells were lysed, and WB was performed using the antibodies indicated. B) Quantification of Pax7

protein levels from A. The Pax7 levels are not recuperated to vehicle levels in cells treated with Imatinib plus MG132 (Imat+MG132 bar, n=3). C) Quantification of MyoD levels from A. The MyoD protein levels are recuperated to vehicle levels in cells treated with Imatinib plus MG132 (Imat+MG132 bar, n=3). D) Pax7 ubiquitination does not increase with Imatinib treatment. C2C12 cells in PM were transfected with plasmids encoding C-terminus fragment of Venus Fluorescent protein (VC155) fused to Pax7 and N-terminus fragment of Venus (VN155-I152L) fused to ubiquitin. As control of transfection, Gap43-mRFP was used (Arrows shows transfected cells). Then cells were treated with vehicle or Imatinib 10 μM for 24 hours. Scale bar: 10 μm. The percentage of BiFC positive cells was determined (Right graph). Veh mean: 46.34±2.56%; Imat mean: 56.77±2.32% (n=3, P-value > 0.05, Mann-Whitney test, ns: not significant). E) BiFC cFos:Jun positive control. Cells were transfected with bFos fused to VC155 and bJun fused to VN155-I152L. Scale bar: 10 μm.
